# Association of the Glutathione S-Transferase M1, T1 Polymorphisms with Cancer: Evidence from a Meta-Analysis

**DOI:** 10.1371/journal.pone.0078707

**Published:** 2013-11-08

**Authors:** Jianzheng Fang, Shangqian Wang, Shengli Zhang, Shifeng Su, Zhen Song, Yunfei Deng, Hongqing Cui, Hainan Wang, Yi Zhang, Jian Qian, Jinbao Gu, Bianjiang Liu, Pengchao Li, Rui Zhang, Xinnong Liu, Zengjun Wang

**Affiliations:** 1 State Key Laboratory of Reproductive Medicine, Department of Urology, The First Affiliated Hospital of Nanjing Medical University, Nanjing, China; 2 Department of Neurosurgery, The First Affiliated Hospital of Nanjing Medical University, Nanjing, China; 3 Department of General Surgery, Qilu Hospital, Shandong University, Jinan,China; University Medical Center Utrecht, The Netherlands

## Abstract

**Background:**

Glutathione S-transferases (GSTs) are a family of multifunctional enzymes that are involved in the metabolism of many xenobiotics, including a wide range of environmental carcinogens. While the null genotypes in *GSTM*1 and *GSTT1* have been implicated in tumorigenesis, it remains inconsistent and inconclusive. Herein, we aimed to assess the possible associations of the *GSTM1* and *GSTT1* null genotype in cancer risks.

**Methods:**

A meta-analysis based on 506 case-control studies was performed. Odds ratios (OR) with corresponding 95% confidence intervals (CIs) were used to assess the association.

**Results:**

The null genotypes of *GSTM1* and *GSTT1* polymorphisms were associated with a significantly increased risk in cancer (for *GSTM1*: OR = 1.17; 95%CI = 1.14–1.21; for *GSTT1*: OR = 1.16; 95%CI = 1.11–1.21, respectively). When the analysis was performed based on their smoking history, the risk associated of *GSTM1* null and *GSTT1* null genotypes with cancer is further increased (for *GSTM*1: OR = 2.66; 95%CI = 2.19–3.24; for *GSTT1*: OR = 2.46; 95%CI = 1.83–3.32, respectively).

**Conclusions:**

These findings indicate that *GSTM1* and *GSTT1* polymorphisms may play critical roles in the development of cancer, especially in smokers.

## Introduction

Glutathione S-transferases (GSTs) are a superfamily of phase II drug-metabolizing enzymes that are involved in the metabolism of many xenobiotics, including a variety of environmental carcinogens by catalyzing the conjugation of glutathione to electrophilic compounds [1]. GSTs also play an vital role in modulating the induction of other enzymes and proteins for cellular functions, for example DNA repair, and are therefore important in maintaining genomic integrity [1]. Cytoplasmic GSTs are classified into eight subfamilies: alpha, kappa, mu, omega, pi, sigma, theta, and zeta [2]. Previous studies showed that a homozygous deletion or null genotype, at either the *GSTM1* locus or the *GSTT1* locus resulted in enzyme function loss, and thus it was hypothesized to be related to the susceptibility to cancer [1], [3]. Although some genetic variants in several of the GST gene families have been identified, most attentions have been focused on *GSTM1* (encoding the mu class) and *GSTT1* (encoding the theta class). *GSTM1* and *GSTT1* genes have a common variant of homozygous deletion (null genotype), which increases vulnerability to cytogenetic damage [4]. Over the past decades, an increasing number of studies have investigated the association between *GSTM1* or GSTT1 polymorphisms and cancer risk in human. Given the biological function of GSTs, many epidemiological studies have focused on the association of *GSTM1* and *GSTT1* polymorphisms with cancer risk in human. However, the results from different studies are to some extent divergent, which may be attributing to limitations in individual studies [5]–[12]. Hence, we performed a meta-analysis with subgroup analysis of eligible studies to acquire more accurate estimation of the association of *GSTM1* or *GSTT1* with cancer risk.

## Materials and Methods

### Identification and Eligibility of Relevant Studies

All case-control studies on the association of the *GSTM1* null or *GSTT1* null polymorphisms with cancer risk published up to February 1, 2013 were identified through comprehensive searches using the PubMed database with the following terms and keywords: “*GSTM1*”, “glutathione S-transferase M1”, “*GSTT1*”, “glutathione S-transferase T1”and “polymorphism”, “variation”, “mutation” and in combination with “cancer”, “tumor” and “carcinoma”. The search was limited to human studies and language in English.

### Inclusion Criteria

The following criteria were used for the study selection: (a) a case–control study evaluating at least one of these two polymorphisms (*GSTM1* and *GSTT1*) and cancer risk; (b) using a case-control design; (c) no overlapping data. For the same or overlapping data in the studies published by the same investigators, we selected the most recent study with a larger number of population; (d) sufficient data for estimating an odds ratio (OR) with 95% confidence interval (95% CI). The exclusion criteria are as follows: (a) not for cancer research; (b) review articles; (c) reports without usable data; (d) duplicate publications.

### Data Extraction

Information was carefully extracted from all the eligible publications independently by two researchers (JZ F and SQ W) according to the inclusion criteria listed above. For conflicting evaluation, a consensus was reached by a third reviewer SLZ. The following data were collected from each study (Checklist S1), (Reference in File S1): first author’s name, publication date, country, ethnicity, cancer type, genotyping method, source of controls (population-based [PB] or hospital-based [HB] controls), total numbers of cases and controls and number of cases and controls for *GSTM1* or *GSTT1* polymorphism. Different ethnic descents were categorized as Caucasian, Asian, African and Mixed. Meanwhile, different case-control groups in one study were considered as independent studies.

### Statistical Methods

The strength of association between either *GSTM1* or *GSTT1* polymorphisms and cancer risk was measured by ORs with 95% confidence intervals (CIs). The percentage weight determined by the precision of its estimate of effect and in the statistical software in STATA, is equal to the inverse of the variance. The risks (ORs) of cancer associated with the *GSTM*1 or *GSTT*1 polymorphism were estimated for each study. In our study, the prescience of either *GSTM*1 present or *GSTT1* present was considered the reference genotype. Stratified analyses were also performed by cancer types (if the cancer type contained with less than three individual studies, it was combined and classified as a group of other cancers), ethnicity, source of controls and sample size (subjects≥500 in both case and control groups or not). Study-specific ORs comparing null genotype versus present genotype were combined using random-effects model (the DerSimonian and Laird) or fixed-effects model (the Mantel–Haenszel method), which was determined by the Q-test statistics [13], [14]. An estimation of potential publication bias was carried out by the funnel plot, in which the standard error of log (OR) of each study was plotted against its log (OR). An asymmetric plot suggests a possible publication bias. Funnel plot asymmetry was assessed by the method of Egger’s linear regression test, a linear regression approach to measure funnel plot asymmetry on the natural logarithm scale of the OR.

We also performed a meta-analyses to assess the multiplicative interactions between either *GSTM1* or *GSTT1* null polymorphisms and smoking (ever smoking vs. never smoking) because this approach is more powerful than conventional case-control studies when testing for a possible multiplicative interaction under the assumption of independence between either *GSTM1* or *GSTT1* null polymorphisms and smoking in the population. A 2-tailed P value less than 0.05 was considered as significant. All statistical analyses were performed with the Stata software (version 12.1; Stata Corp LP, College Station, TX, USA).

## Results

### Eligible Studies and Meta-analysis Databases

**Figure 1:**
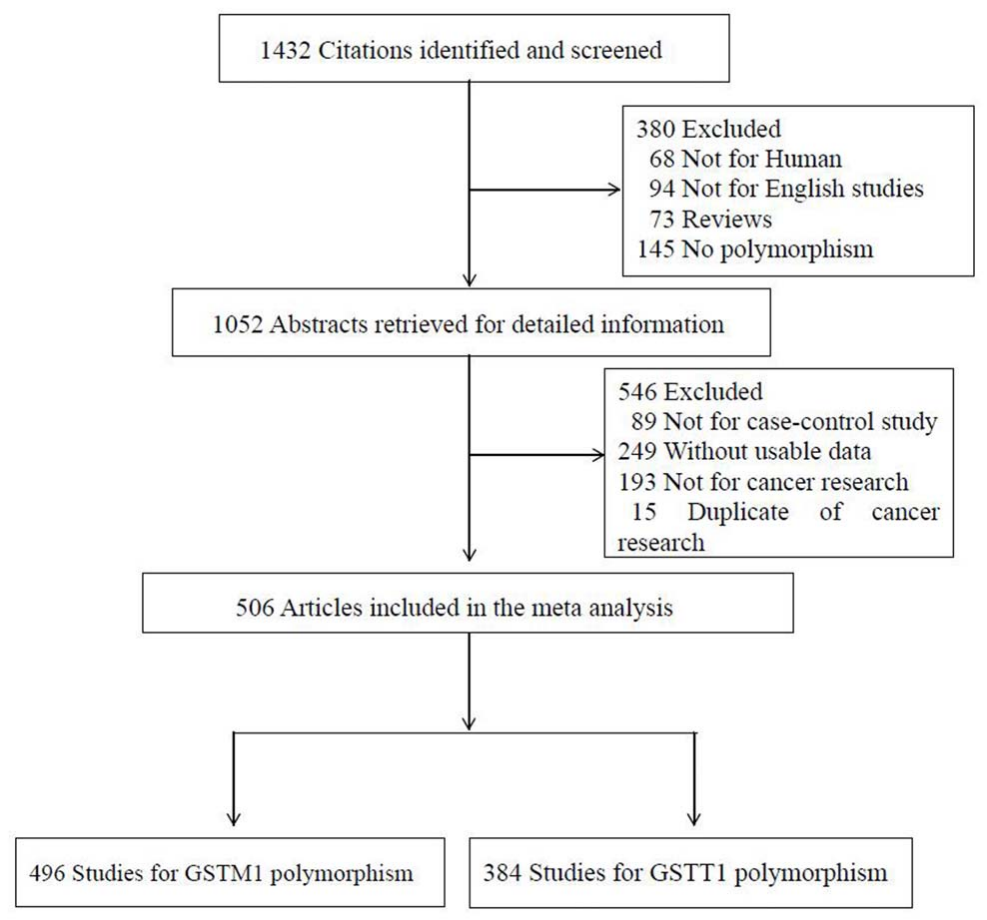
Studies identified with criteria for inclusion and exclusion.

There were 506 studies retrieved on the basis of the search criteria (Fig. 1). A total of 496 studies (113,631cases and 155,007 controls) for *GSTM1* polymorphism and 384 studies (94,740 cases and 126,414 controls) for *GSTT1* polymorphism were selected in the meta-analysis. Study characteristics were summarized in Table S1. For *GSTM*1 polymorphism, there were a total of 254 Caucasians, 176 Asians, 4 Africans and 62 mixed descendants. Controls were selected with matched sex and age, including 348 hospital-based and 148 population-based studies. For *GSTT1* polymorphism, here were 205 Caucasians, 123 Asians,3 Africans and 53 mixed descendants. The sex and age matched controls include 261 hospital-based and 123 population-based studies. Cancers were clinically diagnosed and confirmed by histological or pathologically stated in the original article. Study characteristics are summarized in Table 1.

**Table 1:** Stratification analyses of the GSTM1 and GSTT1 polymorphism on cancer.

Variables	Sample size	GSTM1null vs. GSTM1present		Sample size	GSTT1 null vs. GSTT1 present	
	N^a^	OR(95% CI)	*P*	*N* a	OR(95% CI)	*P*
**Total**		**1.17(1.14–1.21)**	<0.001		**1.16(1.11–1.21)**	<0.001
**Tumor type**						
Hodgkin lymphoma	4	0.54(0.17–1.74)	<0.001	–	–	–
Prostate cancer	34	1.34(1.17–1.54)	<0.001	27	1.05(0.90–1.22)	<0.001
Colorectal cancer	40	1.11(1.04–1.19)	0.001	32	1.13(1.02–1.27)	<0.001
Breast cancer	58	1.12(1.06–1.18)	<0.001	42	1.10(1.02–1.19)	<0.001
Bladder cancer	41	1.38(1.26–1.51)	<0.001	32	1.12(0.98–1.29)	<0.001
Ovarian cancer	8	1.08(0.90–1.29)	0.088	6	1,00(0.86–1.15)	0.909
Chronic myelogeous leukemia	4	0.93(0.66–1.32)	0.158	4	1.57(0.90–2.74)	0.013
Lung cancer	87	1.13(1.06–1.20)	<0.001	52	1.11(1.01–1.22)	<0.001
Acute myeloblastic leukemia	7	1.15(0.84–1.57)	<0.001	7	1.24(0.96–1.61)	0.058
Melanoma	4	0.93(0.79–1.10)	0.745	4	1.08(0.87–1.35)	0.988
Acute lymphoid leukemia	11	1.38(1.08–1.76)	0.001	8	1.07(0.82–1.39)	0.111
Renal cell carcinoma	7	0.98(0.84–1.14)	0.178	8	1.17(0.88–1.53)	<0.001
Gastric cancer	29	1.24(1.08–1.41)	<0.001	25	1.26(1.10–1.44)	0.002
Leukemia	5	1.12(0.82–1.54)	0.051	5	1.29(0.88–1.91)	0.029
Head and Neck cancer	59	1.32(1.17–1.47)	<0.001	46	1.16(1.02–1.33)	<0.001
Endometrial cancer	4	0.96(0.68–1.34)	0.009	4	1.07(0.69–1.66)	0.009
Nasopharyngeal carcinoma	5	1.33(1.13–1.56)	0.614	–	–	–
Cervical cancer	12	1.28(0.97–1.70)	<0.001	11	1.38(0.99–1.93)	<0.001
Esophageal cancer	21	1.12(0.92–1.36)	<0.001	15	0.95(0.81–1.11)	0.228
Hepatocellular carcinoma	13	1.07(0.77–1.49)	<0.001	10	1.23(0.87–1.74)	<0.001
Pancreatic cancer	4	0.94(0.78–1.13)	0.446	–	–	–
Thyroid cancer	9	1.04(0.85–1.28)	0.042	8	1.24(0.82–1.89)	<0.001
Brain tumor	6	1.05(0.85–1.30)	0.041	6	1.10(0.92–1.32)	0.327
Others^b^	24	1.13(0.98–1.31)	0.001	32	1.41(1.12–1.77)	<0.001
**Ethnicity**						
Caucasian	254	1.14(1.09–1.18)	<0.001	205	1.19(1.12–1.25)	<0.001
Asian	176	1.26(1.19–1.33)	<0.001	123	1.14(1.07–1.23)	<0.001
Mixed	62	1.12(1.03–1.23)	<0.001	53	1.10(0.99–1.21)	<0.001
African	4	0.93(0.77–1.12)	0.42	3	0.94(0.53–1.67)	0.013
**Control source**						
Hospital based	348	1.23(1.18–1.29)	<0.001	261	1.20(1.14–1.27)	<0.001
Population based	148	1.07(1.04–1.11)	<0.001	123	1.08(1.03–1.14)	<0.001
**Sample size(both cases and controls)**						
<500	331	1.24(1.19–1.30)	<0.001	248	1.18(1.12–1.25)	<0.001
≥500	165	1.09(1.05–1.14)	<0.001	136	1.13(1.07–1.19)	<0.001

a Number of studies.

b Cancer less than 3 case-control studies.

### Quantitative Synthesis

The relationship between the *GSTM1* or *GSTT1* polymorphisms and the risk of different kinds of cancer are summarized in Table 1.

### GSTM1

Overall, a significant increased risk of cancer is associated with the *GSTM1* polymorphism (null vs. present: OR = 1.17, 95%CI = 1.14–1.21, p<0.001). In the subgroup analysis by ethnicity, the results indicated that individuals with *GSTM1* null genotype had a significantly higher cancer risks among Caucasians (null vs. present: OR = 1.14, 95%CI = 1.09–1.18, p<0.001), Asians (null vs. present: OR = 1.26, 95%CI = 1.19–1.33, p<0.001) and the mixed descendants (null vs. present: OR = 1.12, 95%CI = 1.03–1.23, p<0.001), but not for Africans (null vs. present: OR = 0.93, 95%CI = 0.77–1.12, p = 0.42). This is possibly because that the sample numbers of Africans are relatively small. When restricting the analysis to the source of controls, significant associations were discovered both in the hospital-based source (null vs. present: OR = 1.23, 95%CI = 1.18–1.29, p<0.001) and population-based source (null vs. present: OR = 1.07, 95%CI = 1.04–1.11, p<0.001). In the stratified analysis by cancer types, significant associations (null vs. present) were found in prostate cancer (OR = 1.34, 95%CI = 1.17–1.54, p<0.001), colorectal cancer (OR = 1.11, 95%CI = 1.04–1.19, p = 0.001), breast cancer (OR = 1.12, 95%CI = 1.06–1.18, p<0.001), bladder cancer (OR = 1.38, 95%CI = 1.26–1.51, p<0.001), lung cancer (OR = 1.13, 95%CI = 1.06–1.20, p<0.001), acute lymphocytic leukemia (OR = 1.38, 95%CI = 1.08–1.76, p = 0.001), gastric cancer (OR = 1.24, 95%CI = 1.08–1.41, p<0.001), head and neck cancer (OR = 1.32, 95%CI = 1.17–1.47, p<0.001) and nasopharyngeal carcinoma (OR = 1.33, 95%CI = 1.13–1.56, p<0.001).

### GSTT1

Similar to *GSTM1*, a dramatic increase in the cancer risk is associated with the GSTT1 polymorphism (null vs. present: OR = 1.16, 95%CI = 1.11–1.21, p<0.001). We also performed a subgroup analysis stratified by ethnicity, source of control and cancer type. By ethnicity, statistically significant association was detected in Caucasians (null vs. present: OR = 1.19, 95%CI = 1.12–1.25, p<0.001) and Asians (null vs. present: OR = 1.14, 95%CI = 1.07–1.23, p<0.001). By the source of controls, both hospital-based (null vs. present: OR = 1.20, 95%CI = 1.14–1.27, p<0.001) and population-based control (null vs. present: OR = 1.08, 95%CI = 1.03–1.14, p<0.001) had a statistical significance. In the subgroup analysis stratified by cancer type, significant associations (null vs. present) were found in colorectal cancer (OR = 1.13, 95%CI = 1.02–1.27, p<0.001), breast cancer (OR = 1.10, 95%CI = 1.02–1.19, p<0.001), lung cancer (OR = 1.11, 95%CI = 1.01–1.22, p<0.001), gastric cancer (OR = 1.26, 95%CI = 1.10–1.44, p = 0.002), head and neck cancer (OR = 1.16, 95%CI = 1.02–1.33, p<0.001), and others cancer (OR = 1.41, 95%CI = 1.12–1.77, p<0.001).

### Smoking

Since smoking has been considered as a risk factor for some types of cancers, we asked whether the *GSTM1* or *GSTT1* null genotype further facilitates cancer risk in the population of smokers. To answer this question, we performed an analyses stratified for smoking in order to assess whether the *GSTM1* or *GSTT1* null genotype influence on cancer differently for smokers from non-smokers. Study characteristics were summarized in Table S2. The meta-analysis and pooled analysis indicated that both *GSTM1* null genotype (null vs. present: OR = 2.66; 95%CI = 2.19–3.14) and *GSTT1* null genotype (null vs. present: OR = 2.46; 95%CI = 1.83–3.32) were associated with an increased cancer risk in smokers (Table 2). These results indicate that smoking should be considered to influence the effect of both *GSTM1* and *GSTT1* null genotypes on tumor development.

**Table 2:** Odds ratios for cancer with smoking status combinations of GST genotypes.

Variables	Smoking status	Cases	Controls	OR(95%CI)
**GSTM1 present**	**Non-smokers**	2830	6013	**1(Reference)**
	**Smokers**	5377	6597	2.14(1.80–2.55)
**GSTM1 null**	**Non-smokers**	2916	5512	1.28(1.16–1.41)
	**Smokers**	5719	6304	2.66(2.19–3.24)
**GSTT1 present**	**Non-smokers**	4009	6707	**1(Reference)**
	**Smokers**	7440	8093	2.16(1.72–2.70)
**GSTT1 null**	**Non-smokers**	1575	2314	1.14(0.94–1.40)
	**Smokers**	2501	2919	2.46(1.83–3.32)

### Heterogeneity Analysis

There was significant heterogeneity for *GSTM1* allele contrast (null vs. present: P<0.001), Therefore, we used a meta-regression analysis to explore the source of heterogeneity for homozygote comparison (null vs. present) by Ethnicity, cancer types, source of controls and sample size. We found that the sample size (t = −3.32, P = 0.001) as well as the source of control (t = −3.88, P<0.001) contributed to substantial altered heterogeneity. However, we did not find cancer types (t = −1.4, P = 0.162), or ethnicity (t = −0.06, P = 0.951) contributed to source of heterogeneity. Similarly, we found the source of control (t = −2.03, P = 0.043) contributed to substantial heterogeneity in GSTT1.

### Publication Bias

**Figure 2:**
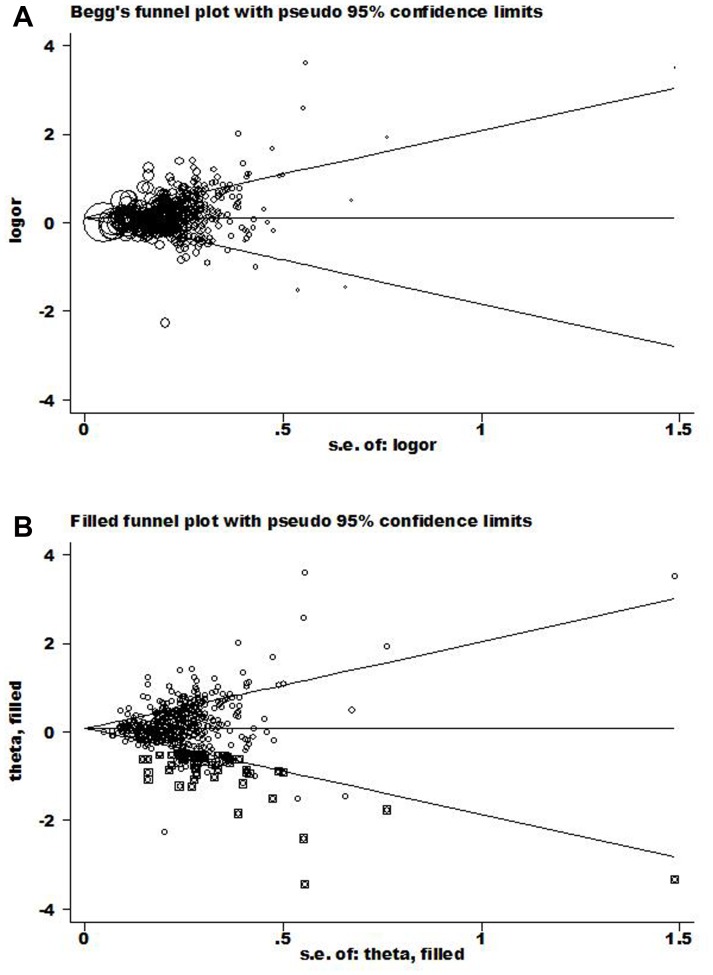
Begg’s funnel plot of publication bias test. (Each point represents a separate study for the indicated association. Log (OR), natural logarithm of OR. Horizontal line, mean effect size.

Begg’s funnel plot and Egger’s test were performed to assess the publication bias. As shown in the Fig. 2, the shapes of the funnel plots seems asymmetrical in both *GSTM1* and *GSTT1* genotypes (*GSTM1*: P<0.001; *GSTT1*: P = 0.005). Thus, the Egger’s test was used to provide statistical evidence of funnel plot symmetry. The both genotypes showed significant publication bias (*GSTM1*: t = 4.97, P<0.001; *GSTT1*: t = 2.88, P = 0.004). To adjust this bias, a trim-and-fill method developed by Duval and Tweedie was used to both identify and correct for funnel plot asymmetry arising from publication bias. We filled in the asymmetric outlying part of the funnel after estimating how many studies were in the asymmetric part with the help of Stata software. Results in figure 2 showed that 60 studies should be filled after iterations. We then estimated the true center of the funnel, the true mean, and its 95%CI, based on the filled funnel plot. The OR estimates and 95%CI of *GSTM1* in fixed-effect model before and after trim-and-fill were 1.132, (1.114–1.150) and 1.081, (1.065–1.098). Also, for random-effect model, the results were 1.173, (1.138–1.209) and 1.085, (1.050–1.122). Meta-analysis with or without the trim-and-fill method did not yield any different conclusions, indicating that our results in *GSTM1* were statistically robust. In *GSTT1*, no studies were filled, as a consequence, no changes were observed before or after trim-and-fill method, which also indicates high reliability.

In conclusion, this meta-analysis demonstrates that *GSTM1* and *GSTT1* null genotypes are risk factors in multiple types of cancers. We also identified that smoking further increases the cancer risk, interestingly not only to lung cancers, in people with either *GSTM1* or *GSTT1* null genotypes.

## Discussion

GSTs are the most important parts of phase II superfamily of metabolism enzymes. In humans, there are several GST classes that were encoded by distinct gene families [2]. Among them, *GSTM1* and *GSTT1* should be pointed out because a polymorphic deletion of these genes may influence the enzyme activity, and eventually increased vulnerability to genotoxic damage [15]. GSTs play a major role in cellular antimutagen and antioxidant defense mechanisms, and these enzymes may regulate pathways that prevent damage from several carcinogens. High levels of GSTs have been shown to detoxify several chemical carcinogens efficiently and to protect tissues against DNA damage [16], [17]. Individuals with homozygous deletions of *GSTM1* or *GSTT1* lack GSTs and therefore may be unable to eliminate electrophilic carcinogens efficiently, which may increase the risk of somatic mutations that lead to tumor formation. Based on these backgrounds, the association between *GSTM1* and *GSTT1* has been intensively investigated polymorphisms and risk of a variety of cancer, but the results remain contradictory. The individual studies might have been underpowered to detect the overall effect of polymorphisms on the susceptibility of cancer. Meta-analysis has been considered to be a relative powerful tool to solve this problem by combining the results from independent studies together. To the best of our knowledge, this is the first meta-analysis with the largest and most comprehensive assessment for the relationship between the *GSTM1* and *GSTT1* polymorphisms and the cancer risk.

In the present study, we examined the association between *GSTM1* and *GSTT1* null genotypes and cancer risk and assessed the multiplicative interactions among *GSTM1*, *GSTT1*, and smoking status. Our results demonstrated that these two polymorphisms are significant associated with cancer risk when all studies were pooled together. Stratified analysis by cancer type for these two polymorphisms indicated that the *GSTM1* null genotype was significantly associated with increased cancer risks for prostate cancer, colorectal cancer, breast cancer, bladder cancer, lung cancer, ALL, gastric cancer, head and neck cancer, and nasopharyngeal carcinoma. These results are in agreement with the previous meta-analysis [11], [18]–[25]. Meanwhile, our results indicated that the *GSTT1* null genotype may be a risk factor for colorectal cancer, breast cancer, lung cancer, gastric cancer, head and neck cancer, “others cancers”, and not for prostate cancer, bladder cancer and ALL. But Yang et al [26] and Gong at al [7] results indicated that *GSTT1* null genotype was significantly increased prostate cancer and bladder cancer risk, respectively. Conflicting results might be owing to that our studies with relative small sample sizes may be underpowered for detecting the real association. Larger studies are needed to testify whether the *GSTM1* or *GSTT1* polymorphisms could truly impact on different types of cancer.

In the subgroup analysis by ethnicity suggested that a possible association between the null genotype of *GSTM1* and *GSTT1* with higher risk of cancer in Asians and Caucasians but not in Africans. Butsome studies [6], [7], [18], [27], [28] indicated that there was an obviously difference between either *GSTM1* or *GSTT1* polymorphisms and ethnicity, especially in Asians and Caucasians. For some cancer, the different susceptibility of cancer in Asians and Caucasians perhaps exist, but unlikely for total cancer. In African group, the sample size and numbers of researches were not adequate to assess the association. Other factors such as selection bias may contribute to it. Hence, the results should be interpreted with caution.

We reviewed here published papers where an estimate of gene–smoking interaction between *GSTM1* or *GSTT1* polymorphisms and cancer risk was available. Our study suggests that the *GSTM1* and *GSTT1* null genotype significantly increase the carcinogenic effect in patients with smoking. Tobacco products contain over 3000 compounds, including many carcinogens and procarcinogens [29]. The effect of these compounds on tobacco-related cancer might be mediated by genetic polymorphisms encoding tobacco metabolizing enzymes such as *GSTM1*, *GSTT1* [30]. Wallstrom et al. [31] examined the association of plasma auto-antibodies against the oxidized DNA base derivative 5-hydroxymethyl-2′-deoxyuridine as a biomarker of oxidative stress with the risk factors smoking, related to the genetic state of *GSTM1* and *GSTT1* in a cross-sectional sample (264 men and 280 women) from the population-based Swedish. They found that the current smokers lacking *GSTM1* had higher auto-antibodies titers, compared with non-smokers or persons expressing *GSTM1*, indicating that smoking increase the production of oxidative stress, especially in people carrying *GSTM1* null genotype. Consequently, these populations tend to be more susceptible to gene damage and thus increase the cancer susceptibility.

The strengths of our meta-analysis included: first, a huge number of cases and controls as many as one hundred thousand people were pooled from different studies, which significantly increased statistical power of the analysis; second, studies included in our present meta-analysis strictly met our selection criteria; third, smoking, an important environment factor, was incorporated into to our study, our results demonstrate that smoking may increase cancer susceptibility with *GSTM1*, *GSTT1* null genotype.

Several limitations might be included in this study. Since most of the included studies have conducted on Asians, Caucasians, and a few on Africans, the results must be interpreted with caution. Additionally, a possible publication bias might have been introduced as only published studies written in English that could be searched from Medline database were included. Moreover, our results were just concerned with smoking without adjustment for other risk factors such as age, dietary habit, and drinking status, environmental factors and other variables, which might have caused serious confounding bias. Finally, we should put smoking more detailed analysis. Smoking can be divided into packages per day or active and passive smokers, which may reflect more accurately.

## Conclusions

In conclusion, this meta-analysis demonstrates that *GSTM1* and *GSTT1* null genotypes seem to be risk factors. Nevertheless, large scales, more rigorous designs, especially studies stratified for gene–gene and gene–environment interactions on these two polymorphisms and cancer risk are needed to research, which may eventually lead to better comprehensive understanding of the possible roles in tumorigenesis.

## Supporting Information

Table S1 The genetype frequencies on each studies. A generalized distribution of genetype frequencies on each included studies are listed.
(XLSX)

Table S2 The genetype frequencies on studies of smoking. A generalized distribution of genetype frequencies on each included studies are listed.
(XLSX)

Checklist S1 PRISMA 2009 Checklist.
(DOC)

File S1 The list of references included in this meta-analysis.
(DOCX)
