# Supplementary material for: Association of the Glutathione S-Transferase M1, T1 Polymorphisms with Cancer: Evidence from a Meta-Analysis
Source: PLoS One. 2013 Nov 8;8(11):e78707. doi: 10.1371/journal.pone.0078707 (PMC3826727; doi:10.1371/journal.pone.0078707)
Supplement: File S1 — The list of references included in this meta-analysis. (DOCX) [file pone.0078707.s004.docx]

1 Yri OE, Ekstrøm PO, Hilden V, Gaudernack G, Liestøl K, et al. (2012). "Polymorphisms in genes encoding interleukin-10 and drug metabolizing enzymes GSTP1, GSTT1, GSTA1 and UGT1A1 influence risk and outcome in Hodgkin lymphoma." Leuk Lymphoma **53**(10): 1934-1944.

2 Van Hemelrijck M, Rohrmann S, Steinbrecher A, Kaaks R, Teucher B, et al. (2012). "Heterocyclic aromatic amine [HCA] intake and prostate cancer risk: effect modification by genetic variants." Nutr Cancer **64**(5): 704-713.

3 Rudolph A, Hein R, Hoffmeister M, Försti A, Hemminki K, et al. (2012). "Copy number variations of GSTT1 and GSTM1, colorectal cancer risk and possible effect modification of cigarette smoking and menopausal hormone therapy." Int J Cancer **131**(5): E841-848.

4 Ramalhinho AC, Fonseca-Moutinho JA, Breitenfeld Granadeiro LA. (2012). "Positive association of polymorphisms in estrogen biosynthesis gene, CYP19A1, and metabolism, GST, in breast cancer susceptibility." DNA Cell Biol **31**(6): 1100-1106

5 Ovsiannikov D, Selinski S, Lehmann ML, Blaszkewicz M, Moormann O, et al. (2012). "Polymorphic enzymes, urinary bladder cancer risk, and structural change in the local industry." J Toxicol Environ Health A **75**(8-10): 557-565

6 Oliveira C, Lourenço GJ, Sagarra RA, Derchain SF, Segalla JG, et al. (2012). "Polymorphisms of glutathione S-transferase Mu 1 (GSTM1), Theta 1 (GSTT1), and Pi 1 (GSTP1) genes and epithelial ovarian cancer risk." Dis Markers **33**(3): 155-159.

7 Luo J, Gao YT, Chow WH, Shu XO, Li H, et al. (2012). "Urinary polyphenols, glutathione S-transferases copy number variation, and breast cancer risk: results from the Shanghai women's health study." Mol Carcinog **51**(5): 379-388.

8 Lordelo GS, Miranda-Vilela AL, Akimoto AK, Alves PC, Hiragi CO, et al. (2012). "Association between methylene tetrahydrofolate reductase and glutathione S-transferase M1 gene polymorphisms and chronic myeloid leukemia in a Brazilian population." Genet Mol Res **11**(2): 1013-1026.

9 Li W, Yue W, Zhang L, Zhao X, Ma L, et al. (2012). "Polymorphisms in GSTM1, CYP1A1, CYP2E1, and CYP2D6 are associated with susceptibility and chemotherapy response in non-small-cell lung cancer patients." Lung **190**(1): 91-98.

10 Lesseur C, Gilbert-Diamond D, Andrew AS, Ekstrom RM, Li Z, Kelsey KT, et al. (2012). "A case-control study of polymorphisms in xenobiotic and arsenic metabolism genes and arsenic-related bladder cancer in New Hampshire." Toxicol Lett **210**(1): 100-106.

11 Kiyohara C, Horiuchi T, Takayama K, Nakanishi Y. (2012). "Genetic polymorphisms involved in carcinogen metabolism and DNA repair and lung cancer risk in a Japanese population." J Thorac Oncol **7**(6): 954-962.

12 Kim HN, Kim NY, Yu L, Tran HT, Kim YK, et al. (2012). "Association of GSTT1 polymorphism with acute myeloid leukemia risk is dependent on smoking status." Leuk Lymphoma **53**(4): 681-687

13 Ibarrola-Villava M, Martin-Gonzalez M, Lazaro P, Pizarro A, Lluch A, et al. (2012). "Role of glutathione S-transferases in melanoma susceptibility: association with GSTP1 rs1695 polymorphism." Br J Dermatol **166**(6): 1176-1183.

14 Faber EW, Lourenço GJ, Ortega MM, Lorand-Metze I, De Souza CA, et al. (2012). "Polymorphisms of VEGF, GSTM1 and GSTT1 genes in multiple myeloma risk." Hematol Oncol **30**(2): 105-107.

15 Dzian A, Halasova E, Matakova T, Kavcova E, Smolar M, et al. (2012). "Lung adenocarcinoma and squamous cell carcinoma in association with genetic polymorphisms of GSTs in Slovak population." Neoplasma **59**(2): 160-167.

16 Chauhan PS, Ihsan R, Mishra AK, Yadav DS, Saluja S, et al. (2012). "High order interactions of xenobiotic metabolizing genes and P53 codon 72 polymorphisms in acute leukemia." Environ Mol Mutagen **53**(8): 619-630

17 Catsburg C, Joshi AD, Corral R, Lewinger JP, Koo J, et al. (2012). "Polymorphisms in carcinogen metabolism enzymes, fish intake, and risk of prostate cancer." Carcinogenesis **33**(7): 1352-1359.

18 Bhat G, Bhat A, Wani A, Sadiq N, Jeelani S, et al. (2012). "Polymorphic variation in glutathione-S-transferase genes and risk of chronic myeloid leukaemia in the Kashmiri population." Asian Pac J Cancer Prev **13**(1): 69-73.

19 Atinkaya C, Taspinar M, Sakiragaoglu O, Oz G, Yazici U, et al. (2012). "The effect of CYP1A1, GSTT1 and GSTM1 polymorphisms on the risk of lung cancer: a case-control study." Hum Exp Toxicol **31**(10): 1074-1080.

20 Ahmad ST, Arjumand W, Seth A, Kumar Saini A, Sultana S. (2012). "Impact of glutathione transferase M1, T1, and P1 gene polymorphisms in the genetic susceptibility of North Indian population to renal cell carcinoma." DNA Cell Biol **31**(4): 636-643.

21 Ada AO, Kunak SC, Hancer F, Soydas E, Alpar S,et al. (2012). "Association between GSTM1, GSTT1, and GSTP1 polymorphisms and lung cancer risk in a Turkish population." Mol Biol Rep **39**(5): 5985-5993.

22 Abdel Rahman HA, Khorshied MM, Elazzamy HH, Khorshid OM. et al. (2012). "The link between genetic polymorphism of glutathione-S-transferases, GSTM1, and GSTT1 and diffuse large B-cell lymphoma in Egypt." J Cancer Res Clin Oncol **138**(8): 1363-1368.

23 Zhang AP, Liu BH, Wang L, Gao Y, Li F, et al. (2011). "Glutathione S-transferase gene polymorphisms and risk of gastric cancer in a Chinese population." Asian Pac J Cancer Prev **12**(12): 3421-3425.

24 Wang J, Jiang J, Zhao Y, Gajalakshmi V, Kuriki K, et al. (2011). "Genetic polymorphisms of glutathione S-transferase genes and susceptibility to colorectal cancer: a case-control study in an Indian population." Cancer Epidemiol **35**(1): 66-72.

25 Tamaki Y, Arai T, Sugimura H, Sasaki T, Honda M, et al. (2011). "Association between cancer risk and drug-metabolizing enzyme gene (CYP2A6, CYP2A13, CYP4B1, SULT1A1, GSTM1, and GSTT1) polymorphisms in cases of lung cancer in Japan." Drug Metab Pharmacokinet **26**(5): 516-522.

26 Salinas-Sánchez AS, Sánchez-Sánchez F, Donate-Moreno MJ, Rubio-del-Campo A, Gimenez-Bachs JM, et al. (2011). "Polymorphic deletions of the GSTT1 and GSTM1 genes and susceptibility to bladder cancer." BJU Int **107**(11): 1825-1832

27 Safarinejad MR, Shafiei N, Safarinejad SH. (2011). "Glutathione S-transferase gene polymorphisms (GSTM1, GSTT1, GSTP1) and prostate cancer: a case-control study in Tehran, Iran." Prostate Cancer Prostatic Dis **14**(2): 105-113.

28 Rouissi K, Ouerhani S, Hamrita B, Bougatef K, Marrakchi R, et al. (2011). "Smoking and polymorphisms in xenobiotic metabolism and DNA repair genes are additive risk factors affecting bladder cancer in Northern Tunisia." Pathol Oncol Res **17**(4): 879-886.

29 Öztürk T, Kahraman ÖT, Toptaş B, Kisakesen Hİ, Çakalir C, et al. (2011). "The effect of CYP1A1 and GSTM1 gene polymorphisms in bladder cancer development in a Turkish population." In Vivo **25**(4): 663-668.

30 Ouerhani S, Nefzi MA, Menif S, Safra I, Douzi K, et al. (2011). "Influence of genetic polymorphisms of xenobiotic metabolizing enzymes on the risk of developing leukemia in a Tunisian population." Bull Cancer **98**(12): 95-106.

31 Nosheen M, Malik FA, Kayani MA. (2011). "Lack of influence of glutathione S-transferase gene deletions in sporadic breast cancer in Pakistan." Asian Pac J Cancer Prev **12**(7): 1749-1752.

32 Naushad SM, Reddy CA, Rupasree Y, Pavani A, Digumarti RR, et al. (2011). "Cross-talk between one-carbon metabolism and xenobiotic metabolism: implications on oxidative DNA damage and susceptibility to breast cancer." Cell Biochem Biophys **61**(3): 715-723.

33 Matejcic M, Li D, Prescott NJ, Lewis CM, Mathew CG, et al. (2011). "Association of a deletion of GSTT2B with an altered risk of oesophageal squamous cell carcinoma in a South African population: a case-control study." PLoS One **6**(12): e29366.

34  Masood N, Kayani MA, Malik FA, Mahjabeen I, Baig RM, et al. (2011). "Genetic variation in carcinogen metabolizing genes associated with oral cancer in pakistani population." Asian Pac J Cancer Prev **12**(2): 491-495.

35 Mandegary A, Rostami S, Alimoghaddam K, Ghavamzadeh A, Ghahremani MH. (2011). "Gluthatione-S-transferase T1-null genotype predisposes adults to acute promyelocytic leukemia; a case-control study." Asian Pac J Cancer Prev **12**(5): 1279-1282.

36 Luo YP, Chen HC, Khan MA, Chen FZ, Wan XX, et al. (2011). "Genetic polymorphisms of metabolic enzymes-CYP1A1, CYP2D6, GSTM1, and GSTT1, and gastric carcinoma susceptibility." Tumour Biol **32**(1): 215-222.

37 Lu XF, Yang WL, Wan ZH, Li J, Bi ZG. (2011). "Glutathione S-transferase polymorphisms and bone tumor risk in China." Asian Pac J Cancer Prev **12**(12): 3357-3360.

38  Lourenço GJ, Silva EF, Rinck-Junior JA, Chone CT, Lima CS. (2011). "CYP1A1, GSTM1 and GSTT1 polymorphisms, tobacco and alcohol status and risk of head and neck squamous cell carcinoma." Tumour Biol **32**(6): 1209-1215.

39 Kumar V, Yadav CS, Datta SK, Singh S, Ahmed RS, (2011). "Association of GSTM1 and GSTT1 polymorphism with lipid peroxidation in benign prostate hyperplasia and prostate cancer: a pilot study." Dis Markers **30**(4): 163-169.

40 Koutros S, Silverman DT, Baris D, Zahm SH, Morton LM, et al. (2011). "Hair dye use and risk of bladder cancer in the New England bladder cancer study." Int J Cancer **129**(12): 2894-2904

41 Kohno T, Kunitoh H, Mimaki S, Shiraishi K, Kuchiba A, et al. (2011). "Contribution of the TP53, OGG1, CHRNA3, and HLA-DQA1 genes to the risk for lung squamous cell carcinoma." J Thorac Oncol **6**(4): 813-817.

42 Koh WP, Nelson HH, Yuan JM, Van den Berg D, Jin A, et al. (2011). "Glutathione S-transferase (GST) gene polymorphisms, cigarette smoking and colorectal cancer risk among Chinese in Singapore." Carcinogenesis **32**(10): 1507-1511.

43 Karageorgi S, Prescott J, Wong JY, Lee IM, Buring JE, et al. (2011). "GSTM1 and GSTT1 copy number variation in population-based studies of endometrial cancer risk." Cancer Epidemiol Biomarkers Prev **20**(7): 1447-1452.

44 Jiang Y, Li N, Dong P, Zhang N, Sun Y, et al. (2011). "Polymorphisms in GSTM1, GSTTI and GSTP1 and nasopharyngeal cancer in the east of China: a case-control study." Asian Pac J Cancer Prev **12**(11): 3097-3100.

45 Ihsan R, Chauhan PS, Mishra AK, Yadav DS, Kaushal M, et al. (2011). "Multiple analytical approaches reveal distinct gene-environment interactions in smokers and non smokers in lung cancer." PLoS One **6**(12): e29431.

46 Goerlitz D, El Daly M, Abdel-Hamid M, Saleh DA, Goldman L, et al. (2011). "GSTM1, GSTT1 null variants, and GPX1 single nucleotide polymorphism are not associated with bladder cancer risk in Egypt." Cancer Epidemiol Biomarkers Prev **20**(7): 1552-1554.

47 Fortes C, Mastroeni S, Boffetta P, Innocenzi L, Antonelli G, et al. (2011). "Polymorphisms of GSTM1 and GSTT1, sun exposure and the risk of melanoma: a case-control study." Acta Derm Venereol **91**(3): 284-289.

48 Darazy M, Balbaa M, Mugharbil A, Saeed H, Sidani H, et al. (2011). "CYP1A1, CYP2E1, and GSTM1 gene polymorphisms and susceptibility to colorectal and gastric cancer among Lebanese." Genet Test Mol Biomarkers **15**(6): 423-429.

49 Cribb AE, Joy Knight M, Guernsey J, Dryer D, Hender K, et al. (2011). "CYP17, catechol-o-methyltransferase, and glutathione transferase M1 genetic polymorphisms, lifestyle factors, and breast cancer risk in women on Prince Edward Island." Breast J **17**(1): 24-31.

50  Chiyomaru K, Nagano T, Nishigori C. (2011). "Polymorphisms of glutathione S-transferase in skin cancers in a Japanese population." Kobe J Med Sci **57**(1): E11-16.

51 Chan JY, Ugrasena DG, Lum DW, Lu Y, Yeoh AE. (2011). "Xenobiotic and folate pathway gene polymorphisms and risk of childhood acute lymphoblastic leukaemia in Javanese children." Hematol Oncol **29**(3): 116-123.

52  Ashtiani ZO, Hasheminasab SM, Ayati M, Goulian BS, Modarressi MH. (2011). "Are GSTM1, GSTT1 and CAG repeat length of androgen receptor gene polymorphisms associated with risk of prostate cancer in Iranian patients?" Pathol Oncol Res **17**(2): 269-275.

53 Anantharaman D, Samant TA, Sen S, Mahimkar MB. (2011). "Polymorphisms in tobacco metabolism and DNA repair genes modulate oral precancer and cancer risk." Oral Oncol **47**(9): 866-872

54 Yu KD, Fan L, Di GH, Yuan WT, Zheng Y, et al. (2010). "Genetic variants in GSTM3 gene within GSTM4-GSTM2-GSTM1-GSTM5-GSTM3 cluster influence breast cancer susceptibility depending on GSTM1." Breast Cancer Res Treat **121**(2): 485-496.

55 Yeh CC, Lai CY, Hsieh LL, Tang R, Wu FY, et al. (2010). "Protein carbonyl levels, glutathione S-transferase polymorphisms and risk of colorectal cancer." Carcinogenesis **31**(2): 228-233.

56 Yang G, Gao YT, Shu XO, Cai Q, Li GL, et al. (2010). "Isothiocyanate exposure, glutathione S-transferase polymorphisms, and colorectal cancer risk." Am J Clin Nutr **91**(3): 704-711.

57 Yadav DS, Devi TR, Ihsan R, Mishra AK, Kaushal M, et al. (2010). "Polymorphisms of glutathione-S-transferase genes and the risk of aerodigestive tract cancers in the Northeast Indian population." Genet Test Mol Biomarkers **14**(5): 715-723.

58 Ueda M, Toji E, Nunobiki O, Sato N, Izuma S, et al. (2010). "Germline polymorphisms of glutathione-S-transferase GSTM1, GSTT1 and p53 codon 72 in cervical carcinogenesis." Hum Cell **23**(4): 119-125.

59 Timofeeva M, Kropp S, Sauter W, Beckmann L, Rosenberger A, et al. (2010). "Genetic polymorphisms of MPO, GSTT1, GSTM1, GSTP1, EPHX1 and NQO1 as risk factors of early-onset lung cancer." Int J Cancer **127**(7): 1547-1561.

60 Steinbrecher A, Rohrmann S, Timofeeva M, Risch A, Jansen E, et al. (2010). "Dietary glucosinolate intake, polymorphisms in selected biotransformation enzymes, and risk of prostate cancer." Cancer Epidemiol Biomarkers Prev **19**(1): 135-143.

61 Tsuchiya Y, Baez S, Calvo A, Pruyas M, Nakamura K,et al. (2010). "Evidence that genetic variants of metabolic detoxication and cell cycle control are not related to gallbladder cancer risk in Chilean women." Int J Biol Markers **25**(2): 75-78.

62 Soucek P, Susova S, Mohelnikova-Duchonova B, Gromadzinska J, Moraviec-Sztandera A,et al. (2010). "Polymorphisms in metabolizing enzymes and the risk of head and neck squamous cell carcinoma in the Slavic population of the central Europe." Neoplasma **57**(5): 415-421.

63 Singh AP, Pant MC, Ruwali M, Shah PP, Prasad R, et al. (2010). "Polymorphism in cytochrome P450 1A2 and their interaction with risk factors in determining risk of squamous cell lung carcinoma in men." Cancer Biomark **8**(6): 351-359.

64  Sharma R, Ahuja M, Panda NK, Khullar M. et al. (2010). "Combined effect of smoking and polymorphisms in tobacco carcinogen-metabolizing enzymes CYP1A1 and GSTM1 on the head and neck cancer risk in North Indians." DNA Cell Biol **29**(8): 441-448.

65 Sam SS, Thomas V, Reddy KS, Surianarayanan G, Chandrasekaran A. (2010). "Gene-environment interactions associated with CYP1A1 MspI and GST polymorphisms and the risk of upper aerodigestive tract cancers in an Indian population." J Cancer Res Clin Oncol **136**(6): 945-951.

66 Salinas-Souza C, Petrilli AS, de Toledo SR. (2010). "Glutathione S-transferase polymorphisms in osteosarcoma patients." Pharmacogenet Genomics **20**(8): 507-515.

67 Palma S, Novelli F, Padua L, Venuti A, Prignano G, et al. (2010). "Interaction between glutathione-S-transferase polymorphisms, smoking habit, and HPV infection in cervical cancer risk." J Cancer Res Clin Oncol **136**(7): 1101-1109.

68  Palli D, Polidoro S, D'Errico M, Saieva C, Guarrera S, et al. (2010). "Polymorphic DNA repair and metabolic genes: a multigenic study on gastric cancer." Mutagenesis **25**(6): 569-575.

69  Nosheen M, Ishrat M, Malik FA, Baig RM, Kayani MA. (2010). "Association of GSTM1 and GSTT1 gene deletions with risk of head and neck cancer in Pakistan: a case control study." Asian Pac J Cancer Prev **11**(4): 881-885.

70 Nisa H, Kono S, Yin G, Toyomura K, Nagano J, et al. (2010). "Cigarette smoking, genetic polymorphisms and colorectal cancer risk: the Fukuoka Colorectal Cancer Study." BMC Cancer **10**: 274.

71 Nguyen TV, Janssen MJ, van Oijen MG, Bergevoet SM, te Morsche RH, et al. (2010). "Genetic polymorphisms in GSTA1, GSTP1, GSTT1, and GSTM1 and gastric cancer risk in a Vietnamese population." Oncol Res **18**(7): 349-355.

72  Moore LE, Boffetta P, Karami S, Brennan P, Stewart PS, et al. (2010). "Occupational trichloroethylene exposure and renal carcinoma risk: evidence of genetic susceptibility by reductive metabolism gene variants." Cancer Res **70**(16): 6527-6536.

73 Moaven O, Raziee HR, Sima HR, Ganji A, Malekzadeh R, et al. (2010). "Interactions between Glutathione-S-Transferase M1, T1 and P1 polymorphisms and smoking, and increased susceptibility to esophageal squamous cell carcinoma." Cancer Epidemiol **34**(3): 285-290.

74 Masoudi M, Saadat I, Omidvari S, Saadat M. (2010). "Additive effects of genetic variations of xenobiotic detoxification enzymes and DNA repair gene XRCC1 on the susceptibility to breast cancer." Breast Cancer Res Treat **120**(1): 263-265.

75 Malik MA, Upadhyay R, Mittal RD, Zargar SA, Mittal B. (2010). "Association of xenobiotic metabolizing enzymes genetic polymorphisms with esophageal cancer in Kashmir Valley and influence of environmental factors." Nutr Cancer **62**(6): 734-742.

76 Leme CV, Raposo LS, Ruiz MT, Biselli JM, Galbiatti AL, et al. (2010). "[GSTM1 and GSTT1 genes analysis in head and neck cancer patients]." Rev Assoc Med Bras **56**(3): 299-303.

77 Kaushal M, Mishra AK, Raju BS, Ihsan R, Chakraborty A, et al. (2010). "Betel quid chewing as an environmental risk factor for breast cancer." Mutat Res **703**(2): 143-148.

78 Kao CC, Chen MK, Kuo WH, Chen TY, Su SC, et al. (2010). "Influence of glutathione-S-transferase theta (GSTT1) and micro (GSTM1) gene polymorphisms on the susceptibility of hepatocellular carcinoma in Taiwan." J Surg Oncol **102**(4): 301-307.

79 Jin Y, Xu H, Zhang C, Kong Y, Hou Y, et al. (2010). "Combined effects of cigarette smoking, gene polymorphisms and methylations of tumor suppressor genes on non small cell lung cancer: a hospital-based case-control study in China." BMC Cancer **10**: 422.

80 Hlavata I, Vrana D, Smerhovsky Z, Pardini B, Naccarati A, et al. (2010). "Association between exposure-relevant polymorphisms in CYP1B1, EPHX1, NQO1, GSTM1, GSTP1 and GSTT1 and risk of colorectal cancer in a Czech population." Oncol Rep **24**(5): 1347-1353.

81 Gervasini G, San Jose C, Carrillo JA, Benitez J, Cabanillas A. (2010). "GST polymorphisms interact with dietary factors to modulate lung cancer risk: study in a high-incidence area." Nutr Cancer **62**(6): 750-758.

82 Chatzimichalis M, Xenellis J, Tzagaroulakis A, Sarof P, Banis K, et al. (2010). "GSTT1, GSTM1, GSTM3 and NAT2 polymorphisms in laryngeal squamous cell carcinoma in a Greek population." J Laryngol Otol **124**(3): 318-323.

83 Cantor KP, Villanueva CM, Silverman DT, Figueroa JD, Real FX, et al. (2010). "Polymorphisms in GSTT1, GSTZ1, and CYP2E1, disinfection by-products, and risk of bladder cancer in Spain." Environ Health Perspect **118**(11): 1545-1550.

84 Cabral RE, Caldeira-de-Araujo A, Cabral-Neto JB, Costa Carvalho Mda G. (2010). "Analysis of GSTM1 and GSTT1 polymorphisms in circulating plasma DNA of lung cancer patients." Mol Cell Biochem **338**(1-2): 263-269.

85 Asim M, Khan LA, Husain SA, Husain S, Sarma MP, et al. (2010). "Genetic polymorphism of glutathione S transferases M1 and T1 in Indian patients with hepatocellular carcinoma." Dis Markers **28**(6): 369-376.

86 Ashton KA, Proietto A, Otton G, Symonds I, McEvoy M, et al. (2010). "Polymorphisms in genes of the steroid hormone biosynthesis and metabolism pathways and endometrial cancer risk." Cancer Epidemiol **34**(3): 328-337.

87 Altinisik J, Balta ZB, Aydin G, Ulutin T, Buyru N. (2010). "Investigation of glutathione S-transferase M1 and T1 deletions in lung cancer." Mol Biol Rep **37**(1): 263-267.

88 Heidelberg (2010). "Genetic polymorphisms in phase I and phase II enzymes and breast cancer risk associated with menopausal hormone therapy in postmenopausal women." Breast Cancer Res Treat **119**(2): 463-474.

89 Zupa A, Sgambato A, Bianchino G, Improta G, Grieco V, et al. (2009). "GSTM1 and NAT2 polymorphisms and colon, lung and bladder cancer risk: a case-control study." Anticancer Res **29**(5): 1709-1714.

90 Zendehdel K, Bahmanyar S, McCarthy S, Nyren O, Andersson B, et al. (2009). "Genetic polymorphisms of glutathione S-transferase genes GSTP1, GSTM1, and GSTT1 and risk of esophageal and gastric cardia cancers." Cancer Causes Control **20**(10): 2031-2038.

91 Zafereo ME, Sturgis EM, Aleem S, Chaung K, Wei Q, et al. (2009). "Glutathione S-transferase polymorphisms and risk of second primary malignancy after index squamous cell carcinoma of the head and neck." Cancer Prev Res (Phila) **2**(5): 432-439.

92 Yu KD, Di GH, Fan L, Wu J, Hu Z, et al. (2009). "A functional polymorphism in the promoter region of GSTM1 implies a complex role for GSTM1 in breast cancer." FASEB J **23**(7): 2274-2287.

93 Vrana D, Pikhart H, Mohelnikova-Duchonova B, Holcatova I, Strnad R, et al. (2009). "The association between glutathione S-transferase gene polymorphisms and pancreatic cancer in a central European Slavonic population." Mutat Res **680**(1-2): 78-81.

94 Sørensen M, López AG, Andersen PK, Vogel U, Autrup H, et al. (2009). "Stratification for smoking in case-cohort studies of genetic polymorphisms and lung cancer." Lung Cancer **63**(3): 335-340.

95 Song DK, Xing DL, Zhang LR, Li ZX, Liu J, et al. (2009). "Association of NAT2, GSTM1, GSTT1, CYP2A6, and CYP2A13 gene polymorphisms with susceptibility and clinicopathologic characteristics of bladder cancer in Central China." Cancer Detect Prev **32**(5-6): 416-423.

96 Sivonová M, Waczulíková I, Dobrota D, Matáková T, Hatok J, et al. (2009). "Polymorphisms of glutathione-S-transferase M1, T1, P1 and the risk of prostate cancer: a case-control study." J Exp Clin Cancer Res **28**: 32.

97 Saxena A, Dhillon VS, Raish M, Asim M, Rehman S, et al. (2009). "Detection and relevance of germline genetic polymorphisms in glutathione S-transferases (GSTs) in breast cancer patients from northern Indian population." Breast Cancer Res Treat **115**(3): 537-543.

98 Sampaio AC, Morari EC, Bufalo NE, Leite JL, Lima CS,et al. (2009). "Lack of influence of glutathione S-transferase genotype profile on cancer susceptibility in smokers and nonsmokers." Med Sci Monit **15**(1): CR10-15

99 Sampaio AC, Morari EC, Bufalo NE, Leite JL, Lima CS, et al. (2009). "Lack of influence of glutathione S-transferase genotype profile on cancer susceptibility in smokers and nonsmokers." Med Sci Monit **15**(1): CR10-15

100 Rouissi K, Ouerhani S, Marrakchi R, Ben Slama MR, Sfaxi M, et al. (2009). "Combined effect of smoking and inherited polymorphisms in arylamine N-acetyltransferase 2, glutathione S-transferases M1 and T1 on bladder cancer in a Tunisian population." Cancer Genet Cytogenet **190**(2): 101-107.

101 Ruwali M, Khan AJ, Shah PP, Singh AP, Pant MC, et al. (2009). "Cytochrome P450 2E1 and head and neck cancer: interaction with genetic and environmental risk factors." Environ Mol Mutagen **50**(6): 473-482.

102 Ribrag V, Koscielny S, Casasnovas O, Cazeneuve C, Brice P, Morschhauser F, et al. (2009). "Pharmacogenetic study in Hodgkin lymphomas reveals the impact of UGT1A1 polymorphisms on patient prognosis." Blood **113**(14): 3307-3313.

103 Reding KW, Weiss NS, Chen C, Li CI, Carlson CS, et al. (2009). "Genetic polymorphisms in the catechol estrogen metabolism pathway and breast cancer risk." Cancer Epidemiol Biomarkers Prev **18**(5): 1461-1467.

104 Olivieri EH, da Silva SD, Mendonça FF, Urata YN, Vidal DO, et al. (2009). "CYP1A2*1C, CYP2E1*5B, and GSTM1 polymorphisms are predictors of risk and poor outcome in head and neck squamous cell carcinoma patients." Oral Oncol **45**(9): e73-79.

105 Moy KA, Yuan JM, Chung FL, Wang XL, Van Den Berg D, et al. (2009). "Isothiocyanates, glutathione S-transferase M1 and T1 polymorphisms and gastric cancer risk: a prospective study of men in Shanghai, China." Int J Cancer **125**(11): 2652-2659.

106 McCarty KM, Santella RM, Steck SE, Cleveland RJ, Ahn J, et al. (2009). "PAH-DNA adducts, cigarette smoking, GST polymorphisms, and breast cancer risk." Environ Health Perspect **117**(4): 552-558.

107 Matakova T, Sivonova M, Halasova E, Mistuna D, Dzian A, et al. (2009). "Gene polymorphisms of biotransforming enzymes (GSTs) and their association with lung cancer in the Slovakian population." Eur J Med Res **14 Suppl 4**: 275-279.

108 Masoudi M, Saadat I, Omidvari S, Saadat M. (2009). "Genetic polymorphisms of GSTO2, GSTM1, and GSTT1 and risk of gastric cancer." Mol Biol Rep **36**(4): 781-784.

109 Malik MA, Upadhyay R, Mittal RD, Zargar SA, Modi DR,et al. (2009). "Role of xenobiotic-metabolizing enzyme gene polymorphisms and interactions with environmental factors in susceptibility to gastric cancer in Kashmir Valley." J Gastrointest Cancer **40**(1-2): 26-32.

110 Lavender NA, Benford ML, VanCleave TT, Brock GN, Kittles RA, et al. (2009). "Examination of polymorphic glutathione S-transferase (GST) genes, tobacco smoking and prostate cancer risk among men of African descent: a case-control study." BMC Cancer **9**: 397.

111 Kumar M, Agarwal SK, Goel SK. (2009). "Lung cancer risk in north Indian population: role of genetic polymorphisms and smoking." Mol Cell Biochem **322**(1-2): 73-79.

112 Kraggerud SM, Oldenburg J, Alnaes GI, Berg M, Kristensen VN, et al. (2009). "Functional glutathione S-transferase genotypes among testicular germ cell tumor survivors: associations with primary and post-chemotherapy tumor histology." Pharmacogenet Genomics **19**(10): 751-759.

113 Klinchid J, Chewaskulyoung B, Saeteng S, Lertprasertsuke N, Kasinrerk W, et al. (2009). "Effect of combined genetic polymorphisms on lung cancer risk in northern Thai women." Cancer Genet Cytogenet **195**(2): 143-149.

114 Imaizumi T, Higaki Y, Hara M, Sakamoto T, Horita M, et al. (2009). "Interaction between cytochrome P450 1A2 genetic polymorphism and cigarette smoking on the risk of hepatocellular carcinoma in a Japanese population." Carcinogenesis **30**(10): 1729-1734.

115 Grando JP, Kuasne H, Losi-Guembarovski R, Sant'ana Rodrigues I, Matsuda HM, et al. (2009). "Association between polymorphisms in the biometabolism genes CYP1A1, GSTM1, GSTT1 and GSTP1 in bladder cancer." Clin Exp Med **9**(1): 21-28.

116 Epplein M, Wilkens LR, Tiirikainen M, Dyba M, Chung FL, et al. (2009). "Urinary isothiocyanates; glutathione S-transferase M1, T1, and P1 polymorphisms; and risk of colorectal cancer: the Multiethnic Cohort Study." Cancer Epidemiol Biomarkers Prev **18**(1): 314-320.

117 Curtin K, Samowitz WS, Wolff RK, Herrick J, Caan BJ, et al. (2009). "Somatic alterations, metabolizing genes and smoking in rectal cancer." Int J Cancer **125**(1): 158-164.

118 Cote ML, Yoo W, Wenzlaff AS, Prysak GM, Santer SK, et al. (2009). "Tobacco and estrogen metabolic polymorphisms and risk of non-small cell lung cancer in women." Carcinogenesis **30**(4): 626-635.

119 Carpenter CL, Yu MC, London SJ. (2009). "Dietary isothiocyanates, glutathione S-transferase M1 (GSTM1), and lung cancer risk in African Americans and Caucasians from Los Angeles County, California." Nutr Cancer **61**(4): 492-499.

120 Amtha R, Ching CS, Zain R, Razak IA, Basuki B,et al. (2009). "GSTM1, GSTT1 and CYP1A1 polymorphisms and risk of oral cancer: a case-control study in Jakarta, Indonesia." Asian Pac J Cancer Prev **10**(1): 21-26.

121 Altayli E, Gunes S, Yilmaz AF, Goktas S, Bek Y. (2009). "CYP1A2, CYP2D6, GSTM1, GSTP1, and GSTT1 gene polymorphisms in patients with bladder cancer in a Turkish population." Int Urol Nephrol **41**(2): 259-266.

122 Al-Moundhri MS, Alkindy M, Al-Nabhani M, Al-Bahrani B, Burney IA, et al. (2009). "Combined polymorphism analysis of glutathione S-transferase M1/G1 and interleukin-1B (IL-1B)/interleukin 1-receptor antagonist (IL-1RN) and gastric cancer risk in an Omani Arab Population." J Clin Gastroenterol **43**(2): 152-156.

123 Van Emburgh BO, Hu JJ, Levine EA, Mosley LJ, Perrier ND, et al. (2008). "Polymorphisms in CYP1B1, GSTM1, GSTT1 and GSTP1, and susceptibility to breast cancer." Oncol Rep **19**(5): 1311-1321.

124 Torresan C, Oliveira MM, Torrezan GT, de Oliveira SF, Abuázar CS, et al. (2008). "Genetic polymorphisms in oestrogen metabolic pathway and breast cancer: a positive association with combined CYP/GST genotypes." Clin Exp Med **8**(2): 65-71.

125 Taspinar M, Aydos SE, Comez O, Elhan AH, Karabulut HG, et al. (2008). "CYP1A1, GST gene polymorphisms and risk of chronic myeloid leukemia." Swiss Med Wkly **138**(1-2): 12-17.

126 Syamala VS, Sreeja L, Syamala V, Raveendran PB, Balakrishnan R, et al. (2008). "Influence of germline polymorphisms of GSTT1, GSTM1, and GSTP1 in familial versus sporadic breast cancer susceptibility and survival." Fam Cancer **7**(3): 213-220.

127 Suneetha KJ, Nancy KN, Rajalekshmy KR, Sagar TG, Rajkumar T. (2008). "Role of GSTM1 (Present/Null) and GSTP1 (Ile105Val) polymorphisms in susceptibility to acute lymphoblastic leukemia among the South Indian population." Asian Pac J Cancer Prev **9**(4): 733-736.

128 Sobti RC, Kaur P, Kaur S, Janmeja AK, Jindal SK, et al. (2008). "Combined effect of GSTM1, GSTT1 and GSTP1 polymorphisms on histological subtypes of lung cancer." Biomarkers **13**(3): 282-295.

129 Siraj AK, Ibrahim M, Al-Rasheed M, Abubaker J, Bu R,et al. (2008). "Polymorphisms of selected xenobiotic genes contribute to the development of papillary thyroid cancer susceptibility in Middle Eastern population." BMC Med Genet **9**: 61.

130 Singh M, Shah PP, Singh AP, Ruwali M, Mathur N, et al. (2008). "Association of genetic polymorphisms in glutathione S-transferases and susceptibility to head and neck cancer." Mutat Res **638**(1-2): 184-194.

131 Singh H, Sachan R, Devi S, Pandey SN, Mittal B. (2008). "Association of GSTM1, GSTT1, and GSTM3 gene polymorphisms and susceptibility to cervical cancer in a North Indian population." Am J Obstet Gynecol **198**(3): 303 e301-306.

132 Shao J, Gu M, Zhang Z, Xu Z, Hu Q, et al. (2008). "Genetic variants of the cytochrome P450 and glutathione S-transferase associated with risk of bladder cancer in a south-eastern Chinese population." Int J Urol **15**(3): 216-221.

133 Shah PP, Singh AP, Singh M, Mathur N, Pant MC, et al. (2008). "Interaction of cytochrome P4501A1 genotypes with other risk factors and susceptibility to lung cancer." Mutat Res **639**(1-2): 1-10.

134 Sakoda LC, Blackston CR, Xue K, Doherty JA, Ray RM, et al. (2008). "Glutathione S-transferase M1 and P1 polymorphisms and risk of breast cancer and fibrocystic breast conditions in Chinese women." Breast Cancer Res Treat **109**(1): 143-155.

135 Reszka E, Czekaj P, Adamska J, Wasowicz W. (2008). "Relevance of glutathione S-transferase M1 and cytochrome P450 1A1 genetic polymorphisms to the development of head and neck cancers." Clin Chem Lab Med **46**(8): 1090-1096.

136 Rajkumar T, Samson M, Rama R, Sridevi V, Mahji U, et al. (2008). "TGFbeta1 (Leu10Pro), p53 (Arg72Pro) can predict for increased risk for breast cancer in south Indian women and TGFbeta1 Pro (Leu10Pro) allele predicts response to neo-adjuvant chemo-radiotherapy." Breast Cancer Res Treat **112**(1): 81-87.

137 Nishino K, Sekine M, Kodama S, Sudo N, Aoki Y, et al. (2008). "Cigarette smoking and glutathione S-transferase M1 polymorphism associated with risk for uterine cervical cancer." J Obstet Gynaecol Res **34**(6): 994-1001.

138 Müller P, Asher N, Heled M, Cohen SB, Risch A, et al. (2008). "Polymorphisms in transporter and phase II metabolism genes as potential modifiers of the predisposition to and treatment outcome of de novo acute myeloid leukemia in Israeli ethnic groups." Leuk Res **32**(6): 919-929.

139 Majumdar S, Mondal BC, Ghosh M, Dey S, Mukhopadhyay A, et al. (2008). "Association of cytochrome P450, glutathione S-transferase and N-acetyl transferase 2 gene polymorphisms with incidence of acute myeloid leukemia." Eur J Cancer Prev **17**(2): 125-132.

140 Losi-Guembarovski R, Cólus IM, De Menezes RP, Poliseli F, Chaves VN, et al. (2008). "Lack of association among polymorphic xenobiotic-metabolizing enzyme genotypes and the occurrence and progression of oral carcinoma in a Brazilian population." Anticancer Res **28**(2A): 1023-1028.

141 Li M, Guan TY, Li Y, Na YQ.. (2008). "Polymorphisms of GSTM1 and CYP1A1 genes and their genetic susceptibility to prostate cancer in Chinese men." Chin Med J (Engl) **121**(4): 305-308.

142 Lemos MC, Coutinho E, Gomes L, Carrilho F, Rodrigues F, et al. (2008). "Combined GSTM1 and GSTT1 null genotypes are associated with a lower risk of papillary thyroid cancer." J Endocrinol Invest **31**(6): 542-545.

143 Kiran M, Chawla YK, Kaur J. (2008). "Glutathione-S-transferase and microsomal epoxide hydrolase polymorphism and viral-related hepatocellular carcinoma risk in India." DNA Cell Biol **27**(12): 687-694.

144 Karami S, Boffetta P, Rothman N, Hung RJ, Stewart T,et al. (2008). "Renal cell carcinoma, occupational pesticide exposure and modification by glutathione S-transferase polymorphisms." Carcinogenesis **29**(8): 1567-1571.

145 Kang SH, Kim TY, Kim HY, Yoon JH, Cho HI,et al. (2008). "Protective role of CYP1A1*2A in the development of multiple myeloma." Acta Haematol **119**(1): 60-64.

146 Hsu LI, Chiu AW, Huan SK, Chen CL, Wang YH, et al. (2008). "SNPs of GSTM1, T1, P1, epoxide hydrolase and DNA repair enzyme XRCC1 and risk of urinary transitional cell carcinoma in southwestern Taiwan." Toxicol Appl Pharmacol **228**(2): 144-155.

147 Honma HN, De Capitani EM, Perroud MW Jr, Barbeiro AS, Toro IF,et al. (2008). "Influence of p53 codon 72 exon 4, GSTM1, GSTT1 and GSTP1*B polymorphisms in lung cancer risk in a Brazilian population." Lung Cancer **61**(2): 152-162.

148 Hernández A, Xamena N, Surrallés J, Galofré P, Velázquez A, et al. (2008). "Role of GST and NAT2 polymorphisms in thyroid cancer." J Endocrinol Invest **31**(11): 1025-1031.

149 Hatagima A, Costa EC, Marques CF, Koifman RJ, Boffetta P, et al. (2008). "Glutathione S-transferase polymorphisms and oral cancer: a case-control study in Rio de Janeiro, Brazil." Oral Oncol **44**(2): 200-207.

150 Harth V, Schafer M, Abel J, Maintz L, Neuhaus T, Besuden M, et al. (2008). "Head and neck squamous-cell cancer and its association with polymorphic enzymes of xenobiotic metabolism and repair." J Toxicol Environ Health A **71**(13-14): 887-897.

151 Guo X, O'Brien SJ, Zeng Y, Nelson GW, Winkler CA. (2008). "GSTM1 and GSTT1 gene deletions and the risk for nasopharyngeal carcinoma in Han Chinese." Cancer Epidemiol Biomarkers Prev **17**(7): 1760-1763.

152 Gates MA, Tworoger SS, Terry KL, Titus-Ernstoff L, Rosner B, et al. (2008). "Talc use, variants of the GSTM1, GSTT1, and NAT2 genes, and risk of epithelial ovarian cancer." Cancer Epidemiol Biomarkers Prev **17**(9): 2436-2444.

153 de Carvalho CR, da Silva ID, Pereira JS, de Souza NC, Focchi GR, et al. (2008). "Polymorphisms of p53, GSTM1 and GSTT1, and HPV in uterine cervix adenocarcinoma." Eur J Gynaecol Oncol **29**(6): 590-593.

154 Csejtei A, Tibold A, Varga Z, Koltai K, Ember A, et al. (2008). "GSTM, GSTT and p53 polymorphisms as modifiers of clinical outcome in colorectal cancer." Anticancer Res **28**(3B): 1917-1922.

155 Buch SC, Nazar-Stewart V, Weissfeld JL, Romkes M. (2008). "Case-control study of oral and oropharyngeal cancer in whites and genetic variation in eight metabolic enzymes." Head Neck **30**(9): 1139-1147.

156 Boccia S, Cadoni G, Sayed-Tabatabaei FA, Volante M, Arzani D,et al. (2008). "CYP1A1, CYP2E1, GSTM1, GSTT1, EPHX1 exons 3 and 4, and NAT2 polymorphisms, smoking, consumption of alcohol and fruit and vegetables and risk of head and neck cancer." J Cancer Res Clin Oncol **134**(1): 93-100.

157 Al-Dayel F, Al-Rasheed M, Ibrahim M, Bu R, Bavi P, et al. (2008). "Polymorphisms of drug-metabolizing enzymes CYP1A1, GSTT and GSTP contribute to the development of diffuse large B-cell lymphoma risk in the Saudi Arabian population." Leuk Lymphoma **49**(1): 122-129.

158 Zhao H, Lin J, Grossman HB, Hernandez LM, Dinney CP, et al. (2007). "Dietary isothiocyanates, GSTM1, GSTT1, NAT2 polymorphisms and bladder cancer risk." Int J Cancer **120**(10): 2208-2213.

159 Yoshida K, Osawa K, Kasahara M, Miyaishi A, Nakanishi K, et al. (2007). "Association of CYP1A1, CYP1A2, GSTM1 and NAT2 gene polymorphisms with colorectal cancer and smoking." Asian Pac J Cancer Prev **8**(3): 438-444.

160 Yeh CC, Sung FC, Tang R, Chang-Chieh CR, Hsieh LL. (2007). "Association between polymorphisms of biotransformation and DNA-repair genes and risk of colorectal cancer in Taiwan." J Biomed Sci **14**(2): 183-193.

161 Yang M, Choi Y, Hwangbo B, Lee JS. (2007). "Combined effects of genetic polymorphisms in six selected genes on lung cancer susceptibility." Lung Cancer **57**(2): 135-142.

162 Wideroff L, Vaughan TL, Farin FM, Gammon MD, Risch H,et al. (2007). "GST, NAT1, CYP1A1 polymorphisms and risk of esophageal and gastric adenocarcinomas." Cancer Detect Prev **31**(3): 233-236.

163 Suzen HS, Guvenc G, Turanli M, Comert E, Duydu Y, et al. (2007). "The role of GSTM1 and GSTT1 polymorphisms in head and neck cancer risk." Oncol Res **16**(9): 423-429.

164 Steck SE, Gaudet MM, Britton JA, Teitelbaum SL, Terry MB, et al. (2007). "Interactions among GSTM1, GSTT1 and GSTP1 polymorphisms, cruciferous vegetable intake and breast cancer risk." Carcinogenesis **28**(9): 1954-1959

165 Spurdle AB, Chang JH, Byrnes GB, Chen X, Dite GS, et al. (2007). "A systematic approach to analysing gene-gene interactions: polymorphisms at the microsomal epoxide hydrolase EPHX and glutathione S-transferase GSTM1, GSTT1, and GSTP1 loci and breast cancer risk." Cancer Epidemiol Biomarkers Prev **16**(4): 769-774.

166 Soya SS, Vinod T, Reddy KS, Gopalakrishnan S, Adithan C. (2007). "Genetic polymorphisms of glutathione-S-transferase genes (GSTM1, GSTT1 and GSTP1) and upper aerodigestive tract cancer risk among smokers, tobacco chewers and alcoholics in an Indian population." Eur J Cancer **43**(18): 2698-2706.

**167** Sørensen M, Raaschou-Nielsen O, Brasch-Andersen C, Tjønneland A, Overvad K, et al. (2007). "Interactions between GSTM1, GSTT1 and GSTP1 polymorphisms and smoking and intake of fruit and vegetables in relation to lung cancer." Lung Cancer **55**(2): 137-144.

168 Skjelbred CF, Saebø M, Hjartåker A, Grotmol T, Hansteen IL,et al. (2007). "Meat, vegetables and genetic polymorphisms and the risk of colorectal carcinomas and adenomas." BMC Cancer **7**: 228.

169 Schwartzbaum JA, Ahlbom A, Lönn S, Warholm M, Rannug A, et al. (2007). "An international case-control study of glutathione transferase and functionally related polymorphisms and risk of primary adult brain tumors." Cancer Epidemiol Biomarkers Prev **16**(3): 559-565.

170 Samson M, Swaminathan R, Rama R, Sridevi V, Nancy KN, et al. (2007). "Role of GSTM1 (Null/Present), GSTP1 (Ile105Val) and P53 (Arg72Pro) genetic polymorphisms and the risk of breast cancer: a case control study from South India." Asian Pac J Cancer Prev **8**(2): 253-257.

171 Rossini A, Rapozo DC, Soares Lima SC, Guimarães DP, Ferreira MA, et al. (2007). "Polymorphisms of GSTP1 and GSTT1, but not of CYP2A6, CYP2E1 or GSTM1, modify the risk for esophageal cancer in a western population." Carcinogenesis **28**(12): 2537-2542.

172 Osawa Y, Osawa KK, Miyaishi A, Higuchi M, Tsutou A, et al. (2007). "NAT2 and CYP1A2 polymorphisms and lung cancer risk in relation to smoking status." Asian Pac J Cancer Prev **8**(1): 103-108.

173 Ortega MM, Honma HN, Zambon L, Lorand-Metze I, Costa FF, et al. (2007). "GSTM1 and codon 72 P53 polymorphism in multiple myeloma." Ann Hematol **86**(11): 815-819.

174 Nordgard SH, Ritchie MD, Jensrud SD, Motsinger AA, Alnaes GI, et al. (2007). "ABCB1 and GST polymorphisms associated with TP53 status in breast cancer." Pharmacogenet Genomics **17**(2): 127-136.

175 Murta-Nascimento C, Silverman DT, Kogevinas M, García-Closas M, Rothman N, et al. (2007). "Risk of bladder cancer associated with family history of cancer: do low-penetrance polymorphisms account for the increase in risk?" Cancer Epidemiol Biomarkers Prev **16**(8): 1595-1600.

176 Mössner R, Anders N, König IR, Krüger U, Schmidt D, et al. (2007). "Variations of the melanocortin-1 receptor and the glutathione-S transferase T1 and M1 genes in cutaneous malignant melanoma." Arch Dermatol Res **298**(8): 371-379.

177 Moore LE, Malats N, Rothman N, Real FX, Kogevinas M, et al. (2007). "Polymorphisms in one-carbon metabolism and trans-sulfuration pathway genes and susceptibility to bladder cancer." Int J Cancer **120**(11): 2452-2458.

178 Moore LE, Brennan P, Karami S, Hung RJ, Hsu C, et al. (2007). "Glutathione S-transferase polymorphisms, cruciferous vegetable intake and cancer risk in the Central and Eastern European Kidney Cancer Study." Carcinogenesis **28**(9): 1960-1964.

179 Mallick S, Romana M, Blanchet P, Multigner L. (2007). "GSTM1 and GSTT1 polymorphisms and the risk of prostate cancer in a Caribbean population of African descent." Urology **69**(6): 1165-1169.

180 Leite JL, Morari EC, Granja F, Campos GM, Guilhen AC, et al. (2007). "Influence of the glutathione s-transferase gene polymorphisms on the susceptibility to basal cell skin carcinoma." Rev Med Chil **135**(3): 301-306.

181 Landi S, Gemignani F, Neri M, Barale R, Bonassi S, et al. (2007). "Polymorphisms of glutathione-S-transferase M1 and manganese superoxide dismutase are associated with the risk of malignant pleural mesothelioma." Int J Cancer **120**(12): 2739-2743.

182 Jiao L, Bondy ML, Hassan MM, Chang DZ, Abbruzzese JL,et al. (2007). "Glutathione S-transferase gene polymorphisms and risk and survival of pancreatic cancer." Cancer **109**(5): 840-848.

183 Jain M, Kumar S, Lal P, Tiwari A, Ghoshal UC, et al. (2007). "Role of GSTM3 polymorphism in the risk of developing esophageal cancer." Cancer Epidemiol Biomarkers Prev **16**(1): 178-181.

184 Eyada TK, El Ghonemy EG, El Ghoroury EA, El Bassyouni SO, El Masry MK. (2007). "Study of genetic polymorphism of xenobiotic enzymes in acute leukemia." Blood Coagul Fibrinolysis **18**(5): 489-495.

185 Edvardsen H, Kristensen VN, Grenaker Alnaes GI, Bøhn M, Erikstein B, et al. (2007). "Germline glutathione S-transferase variants in breast cancer: relation to diagnosis and cutaneous long-term adverse effects after two fractionation patterns of radiotherapy." Int J Radiat Oncol Biol Phys **67**(4): 1163-1171.

186 Cha IH, Park JY, Chung WY, Choi MA, Kim HJ, et al. (2007). "Polymorphisms of CYP1A1 and GSTM1 genes and susceptibility to oral cancer." Yonsei Med J **48**(2): 233-239.

187 Capoluongo E, Almadori G, Concolino P, Bussu F, Santonocito C, et al. (2007). "GSTT1 and GSTM1 allelic polymorphisms in head and neck cancer patients from Italian Lazio Region." Clin Chim Acta **376**(1-2): 174-178.

188 Bu H, Rosdahl I, Holmdahl-Källen K, Sun XF, Zhang H. (2007). "Significance of glutathione S-transferases M1, T1 and P1 polymorphisms in Swedish melanoma patients." Oncol Rep **17**(4): 859-864.

189 Bolufer P, Collado M, Barragán E, Cervera J, Calasanz MJ, et al. (2007). "The potential effect of gender in combination with common genetic polymorphisms of drug-metabolizing enzymes on the risk of developing acute leukemia." Haematologica **92**(3): 308-314

190 Boccia S, Sayed-Tabatabaei FA, Persiani R, Gianfagna F, Rausei S, et al. (2007). "Polymorphisms in metabolic genes, their combination and interaction with tobacco smoke and alcohol consumption and risk of gastric cancer: a case-control study in an Italian population." BMC Cancer **7**: 206.

191 Anantharaman D, Chaubal PM, Kannan S, Bhisey RA, Mahimkar MB. (2007). "Susceptibility to oral cancer by genetic polymorphisms at CYP1A1, GSTM1 and GSTT1 loci among Indians: tobacco exposure as a risk modulator." Carcinogenesis **28**(7): 1455-1462.

191 Yang J, Wu HF, Zhang W, Gu M, Hua LX, et al. (2006). "Polymorphisms of metabolic enzyme genes, living habits and prostate cancer susceptibility." Front Biosci **11**: 2052-2060.

192 Wu SH, Tsai SM, Hou MF, Lin HS, Hou LA, et al. (2006). "Interaction of genetic polymorphisms in cytochrome P450 2E1 and glutathione S-transferase M1 to breast cancer in Taiwanese woman without smoking and drinking habits." Breast Cancer Res Treat **100**(1): 93-98.

193 Sugimura T, Kumimoto H, Tohnai I, Fukui T, Matsuo K, et al. (2006). "Gene-environment interaction involved in oral carcinogenesis: molecular epidemiological study for metabolic and DNA repair gene polymorphisms." J Oral Pathol Med **35**(1): 11-18.

194 Stankov K, Landi S, Gioia-Patricola L, Bonora E, Volante M, et al. (2006). "GSTT1 and M1 polymorphisms in Hurthle thyroid cancer patients." Cancer Lett **240**(1): 76-82.

195 Sobti RC, Kaur S, Kaur P, Singh J, Gupta I, et al. (2006). "Interaction of passive smoking with GST (GSTM1, GSTT1, and GSTP1) genotypes in the risk of cervical cancer in India." Cancer Genet Cytogenet **166**(2): 117-123.

196 Silig Y, Pinarbasi H, Günes S, Ayan S, Bagci H, et al. (2006). "Polymorphisms of CYP1A1, GSTM1, GSTT1, and prostate cancer risk in Turkish population." Cancer Invest **24**(1): 41-45.

197 Sharma A, Mishra A, Das BC, Sardana S, Sharma JK. (2006). "Genetic polymorphism at GSTM1 and GSTT1 gene loci and susceptibility to oral cancer." Neoplasma **53**(4): 309-315.

198 Quiñones LA, Irarrázabal CE, Rojas CR, Orellana CE, Acevedo C, et al. (2006). "Joint effect among p53, CYP1A1, GSTM1 polymorphism combinations and smoking on prostate cancer risk: an exploratory genotype-environment interaction study." Asian J Androl **8**(3): 349-355.

199 Probst-Hensch NM, Sun CL, Van Den Berg D, Ceschi M, Koh WP, et al. (2006). "The effect of the cyclin D1 (CCND1) A870G polymorphism on colorectal cancer risk is modified by glutathione-S-transferase polymorphisms and isothiocyanate intake in the Singapore Chinese Health Study." Carcinogenesis **27**(12): 2475-2482.

200 Pisani P, Srivatanakul P, Randerson-Moor J, Vipasrinimit S, Lalitwongsa S, et al. (2006). "GSTM1 and CYP1A1 polymorphisms, tobacco, air pollution, and lung cancer: a study in rural Thailand." Cancer Epidemiol Biomarkers Prev **15**(4): 667-674.

201 Peters ES, McClean MD, Marsit CJ, Luckett B, Kelsey KT. (2006). "Glutathione S-transferase polymorphisms and the synergy of alcohol and tobacco in oral, pharyngeal, and laryngeal carcinoma." Cancer Epidemiol Biomarkers Prev **15**(11): 2196-2202.

202 Pandey SN, Jain M, Nigam P, Choudhuri G, Mittal B. (2006). "Genetic polymorphisms in GSTM1, GSTT1, GSTP1, GSTM3 and the susceptibility to gallbladder cancer in North India." Biomarkers **11**(3): 250-261.

203 Nock NL, Liu X, Cicek MS, Li L, Macarie F, et al. (2006). "Polymorphisms in polycyclic aromatic hydrocarbon metabolism and conjugation genes, interactions with smoking and prostate cancer risk." Cancer Epidemiol Biomarkers Prev **15**(4): 756-761.

204 McGrath M, Michaud D, De Vivo I. (2006). "Polymorphisms in GSTT1, GSTM1, NAT1 and NAT2 genes and bladder cancer risk in men and women." BMC Cancer **6**: 239.

205 Martínez C, Martín F, Fernández JM, García-Martín E, Sastre J, et al. (2006). "Glutathione S-transferases mu 1, theta 1, pi 1, alpha 1 and mu 3 genetic polymorphisms and the risk of colorectal and gastric cancers in humans." Pharmacogenomics **7**(5): 711-718.

206 Little J, Sharp L, Masson LF, Brockton NT, Cotton SC, et al. (2006). "Colorectal cancer and genetic polymorphisms of CYP1A1, GSTM1 and GSTT1: a case-control study in the Grampian region of Scotland." Int J Cancer **119**(9): 2155-2164.

207 Lee KM, Kang D, Lee SJ, Park SK, Lee KH,et al. (2006). "Interactive effect of genetic polymorphism of glutathione S-transferase M1 and smoking on squamous cell lung cancer risk in Korea." Oncol Rep **16**(5): 1035-1039.

208 Larsen JE, Colosimo ML, Yang IA, Bowman R, Zimmerman PV, et al. (2006). "CYP1A1 Ile462Val and MPO G-463A interact to increase risk of adenocarcinoma but not squamous cell carcinoma of the lung." Carcinogenesis **27**(3): 525-532.

209 Ladero JM, Martínez C, García-Martín E, Ropero P, Briceño O, Villegas A, et al. (2006). "Glutathione S-transferase M1 and T1 genetic polymorphisms are not related to the risk of hepatocellular carcinoma: a study in the Spanish population." Eur J Cancer **42**(1): 73-77.

210 Joseph T, Chacko P, Wesley R, Jayaprakash PG, James FV, et al. (2006). "Germline genetic polymorphisms of CYP1A1, GSTM1 and GSTT1 genes in Indian cervical cancer: associations with tumor progression, age and human papillomavirus infection." Gynecol Oncol **101**(3): 411-417.

211 Jain M, Kumar S, Rastogi N, Lal P, Ghoshal UC, et al. (2006). "GSTT1, GSTM1 and GSTP1 genetic polymorphisms and interaction with tobacco, alcohol and occupational exposure in esophageal cancer patients from North India." Cancer Lett **242**(1): 60-67.

212 Huang K, Sandler RS, Millikan RC, Schroeder JC, North KE, et al. (2006). "GSTM1 and GSTT1 polymorphisms, cigarette smoking, and risk of colon cancer: a population-based case-control study in North Carolina (United States)." Cancer Causes Control **17**(4): 385-394.

213 Ho T, Zhao C, Zheng R, Liu Z, Wei Q, et al. (2006). "Glutathione S-transferase polymorphisms and risk of differentiated thyroid carcinomas: a case-control analysis." Arch Otolaryngol Head Neck Surg **132**(7): 756-761.

214 Gattás GJ, de Carvalho MB, Siraque MS, Curioni OA, Kohler P, et al. (2006). "Genetic polymorphisms of CYP1A1, CYP2E1, GSTM1, and GSTT1 associated with head and neck cancer." Head Neck **28**(9): 819-826.

215 Chen HC, Cao YF, Hu WX, Liu XF, Liu QX, et al. (2006). "Genetic polymorphisms of phase II metabolic enzymes and lung cancer susceptibility in a population of Central South China." Dis Markers **22**(3): 141-152.

216 Casson AG, Zheng Z, Porter GA, Guernsey DL. (2006). "Genetic polymorphisms of microsomal epoxide hydroxylase and glutathione S-transferases M1, T1 and P1, interactions with smoking, and risk for esophageal (Barrett) adenocarcinoma." Cancer Detect Prev **30**(5): 423-431.

217 Bufalo NE, Leite JL, Guilhen AC, Morari EC, Granja F, et al. (2006). "Smoking and susceptibility to thyroid cancer: an inverse association with CYP1A1 allelic variants." Endocr Relat Cancer **13**(4): 1185-1193.

218 Biselli JM, de Angelo Calsaverini Leal RC, Ruiz MT, Goloni-Bertollo EM, Maníglia JV, et al. (2006) GSTM1 polymorphism in cigarette smokers with head and neck squamous cell carcinoma." Braz J Otorhinolaryngol **72**(5): 654-658.

219 Aydin-Sayitoglu M, Hatirnaz O, Erensoy N, Ozbek U. (2006). "Role of CYP2D6, CYP1A1, CYP2E1, GSTT1, and GSTM1 genes in the susceptibility to acute leukemias." Am J Hematol **81**(3): 162-170.

220 Agudo A, Sala N, Pera G, Capellá G, Berenguer A, et al. (2006). "Polymorphisms in metabolic genes related to tobacco smoke and the risk of gastric cancer in the European prospective investigation into cancer and nutrition." Cancer Epidemiol Biomarkers Prev **15**(12): 2427-2434.

221 Agalliu I, Langeberg WJ, Lampe JW, Salinas CA, Stanford JL. (2006). "Glutathione S-transferase M1, T1, and P1 polymorphisms and prostate cancer risk in middle-aged men." Prostate **66**(2): 146-156.

222 Yeh CC, Hsieh LL, Tang R, Chang-Chieh CR, Sung FC. (2005). "Vegetable/fruit, smoking, glutathione S-transferase polymorphisms and risk for colorectal cancer in Taiwan." World J Gastroenterol **11**(10): 1473-1480.

223 Wenzlaff AS, Cote ML, Bock CH, Land SJ, Schwartz AG. (2005). "GSTM1, GSTT1 and GSTP1 polymorphisms, environmental tobacco smoke exposure and risk of lung cancer among never smokers: a population-based study." Carcinogenesis **26**(2): 395-401.

224 Vijayalakshmi K, Vettriselvi V, Krishnan M, Shroff S, Vishwanathan KN, et al. (2005). "Polymorphisms at GSTM1 and GSTP1 gene loci and risk of prostate cancer in a South Indian population." Asian Pac J Cancer Prev **6**(3): 309-314.

225 van der Hel OL, Bueno-de-Mesquita HB, van Gils CH, Roest M, Slothouber B, et al. (2005). "Cumulative genetic defects in carcinogen metabolism may increase breast cancer risk (The Netherlands)." Cancer Causes Control **16**(6): 675-681.

226 Tiwawech D, Srivatanakul P, Karalak A, Ishida T. (2005). "Glutathione S-transferase M1 gene polymorphism in Thai nasopharyngeal carcinoma." Asian Pac J Cancer Prev **6**(3): 270-275.

227 Tamer L, Ateş NA, Ateş C, Ercan B, Elipek T,et al. (2005). "Glutathione S-transferase M1, T1 and P1 genetic polymorphisms, cigarette smoking and gastric cancer risk." Cell Biochem Funct **23**(4): 267-272.

228 Srivastava DS, Mandhani A, Mittal B, Mittal RD. (2005). "Genetic polymorphism of glutathione S-transferase genes (GSTM1, GSTT1 and GSTP1) and susceptibility to prostate cancer in Northern India." BJU Int **95**(1): 170-173.

229 Srivastava DS, Mishra DK, Mandhani A, Mittal B, Kumar A, et al. (2005). "Association of genetic polymorphism of glutathione S-transferase M1, T1, P1 and susceptibility to bladder cancer." Eur Urol **48**(2): 339-344.

230 Sreeja L, Syamala V, Hariharan S, Madhavan J, Devan SC, et al. (2005). "Possible risk modification by CYP1A1, GSTM1 and GSTT1 gene polymorphisms in lung cancer susceptibility in a South Indian population." J Hum Genet **50**(12): 618-627.

231 Sobti RC, Al-Badran AI, Sharma S, Sharma SK, Krishan A, et al. (2005). "Genetic polymorphisms of CYP2D6, GSTM1, and GSTT1 genes and bladder cancer risk in North India." Cancer Genet Cytogenet **156**(1): 68-73.

232 Shen J, Wang RT, Xu YC, Wang LW, Wang XR. (2005). "Interaction models of CYP1A1, GSTM1 polymorphisms and tobacco smoking in intestinal gastric cancer." World J Gastroenterol **11**(38): 6056-6060.

234 Rajagopal R, Deakin M, Fawole AS, Elder JB, Elder J, et al. (2005). "Glutathione S-transferase T1 polymorphisms are associated with outcome in colorectal cancer." Carcinogenesis **26**(12): 2157-2163.

235 Pinarbasi H, Silig Y, Gurelik M. (2005). "Genetic polymorphisms of GSTs and their association with primary brain tumor incidence." Cancer Genet Cytogenet **156**(2): 144-149.

236 Palli D, Saieva C, Gemma S, Masala G, Gomez-Miguel MJ, et al. (2005). "GSTT1 and GSTM1 gene polymorphisms and gastric cancer in a high-risk italian population." Int J Cancer **115**(2): 284-289.

237 Pakakasama S, Mukda E, Sasanakul W, Kadegasem P, Udomsubpayakul U, et al. (2005). "Polymorphisms of drug-metabolizing enzymes and risk of childhood acute lymphoblastic leukemia." Am J Hematol **79**(3): 202-205.

238 Niwa Y, Hirose K, Nakanishi T, Nawa A, Kuzuya K, et al. (2005). "Association of the NAD(P)H: quinone oxidoreductase C609T polymorphism and the risk of cervical cancer in Japanese subjects." Gynecol Oncol **96**(2): 423-429.

239 Nan HM, Song YJ, Yun HY, Park JS, Kim H. (2005). "Effects of dietary intake and genetic factors on hypermethylation of the hMLH1 gene promoter in gastric cancer." World J Gastroenterol **11**(25): 3834-3841.

240 Mu LN, Lu QY, Yu SZ, Jiang QW, Cao W,et al. (2005). "Green tea drinking and multigenetic index on the risk of stomach cancer in a Chinese population." Int J Cancer **116**(6): 972-983.

241 Lu XM, Zhang YM, Lin RY, Arzi G, Wang X, et al. (2005). "Relationship between genetic polymorphisms of metabolizing enzymes CYP2E1, GSTM1 and Kazakh's esophageal squamous cell cancer in Xinjiang, China." World J Gastroenterol **11**(24): 3651-3654.

242 Linhares JJ, Da Silva ID, De Souza NC, Noronha EC, Ferraro O, et al. (2005). "Genetic polymorphism of GSTM1 in women with breast cancer and interact with reproductive history and several clinical pathologies." Biol Res **38**(2-3): 273-281.

243 Li H, Chen XL, Li HQ. (2005). "Polymorphism of CYPIA1 and GSTM1 genes associated with susceptibility of gastric cancer in Shandong Province of China." World J Gastroenterol **11**(37): 5757-5762.

244 Lanciotti M, Coco S, Michele PD, Haupt R, Boni L,et al. (2005). "Glutathione S-transferase polymorphisms and susceptibility to neuroblastoma." Pharmacogenet Genomics **15**(6): 423-426.

245 Lai MT, Chen RH, Tsai FJ, Wan L, Chen WC. (2005). "Glutathione S-transferase M1 gene but not insulin-like growth factor-2 gene or epidermal growth factor gene is associated with prostate cancer." Urol Oncol **23**(4): 225-229.

246 Lai KC, Chen WC, Tsai FJ, Li SY, Chou MC, et al. (2005). "Glutathione S-transferase M1 gene null genotype and gastric cancer risk in Taiwan." Hepatogastroenterology **52**(66): 1916-1919.

247 Komiya Y, Tsukino H, Nakao H, Kuroda Y, Imai H,et al. (2005). "Human glutathione S-transferase A1, T1, M1, and P1 polymorphisms and susceptibility to prostate cancer in the Japanese population." J Cancer Res Clin Oncol **131**(4): 238-242.

248 Kim EJ, Jeong P, Quan C, Kim J, Bae SC, et al. (2005). "Genotypes of TNF-alpha, VEGF, hOGG1, GSTM1, and GSTT1: useful determinants for clinical outcome of bladder cancer." Urology **65**(1): 70-75.

249 Karagas MR, Park S, Warren A, Hamilton J, Nelson HH, et al. (2005). "Gender, smoking, glutathione-S-transferase variants and bladder cancer incidence: a population-based study." Cancer Lett **219**(1): 63-69.

250 Hishida A, Terakura S, Emi N, Yamamoto K, Murata M, et al. (2005). "GSTT1 and GSTM1 deletions, NQO1 C609T polymorphism and risk of chronic myelogenous leukemia in Japanese." Asian Pac J Cancer Prev **6**(3): 251-255.

251 Gomes L, Lemos MC, Paiva I, Ribeiro C, Carvalheiro M, et al. (2005). "CYP2D6 genetic polymorphisms are associated with susceptibility to pituitary tumors." Acta Med Port **18**(5): 339-343.

252 Gelatti U, Covolo L, Talamini R, Tagger A, Barbone F, et al. (2005). "N-Acetyltransferase-2, glutathione S-transferase M1 and T1 genetic polymorphisms, cigarette smoking and hepatocellular carcinoma: a case-control study." Int J Cancer **115**(2): 301-306.

253 Gajecka M, Rydzanicz M, Jaskula-Sztul R, Kujawski M, Szyfter W, et al. (2005). "CYP1A1, CYP2D6, CYP2E1, NAT2, GSTM1 and GSTT1 polymorphisms or their combinations are associated with the increased risk of the laryngeal squamous cell carcinoma." Mutat Res **574**(1-2): 112-123.

254 Drummond SN, Gomez RS, Motta Noronha JC, Pordeus IA, Barbosa AA, et al. (2005). "Association between GSTT-1 gene deletion and the susceptibility to oral squamous cell carcinoma in cigarette-smoking subjects." Oral Oncol **41**(5): 515-519.

255 Doherty JA, Weiss NS, Freeman RJ, Dightman DA, Thornton PJ, et al. (2005). "Genetic factors in catechol estrogen metabolism in relation to the risk of endometrial cancer." Cancer Epidemiol Biomarkers Prev **14**(2): 357-366.

256 Deng ZL, Wei YP, Ma Y. (2005). "Polymorphism of glutathione S-transferase mu 1 and theta 1 genes and hepatocellular carcinoma in southern Guangxi, China." World J Gastroenterol **11**(2): 272-274.

257 Covolo L, Gelatti U, Talamini R, Garte S, Trevisi P, Franceschi S, et al. (2005). "Alcohol dehydrogenase 3, glutathione S-transferase M1 and T1 polymorphisms, alcohol consumption and hepatocellular carcinoma (Italy)." Cancer Causes Control **16**(7): 831-838.

258 Clavel J, Bellec S, Rebouissou S, Ménégaux F, Feunteun J, et al. (2005). "Childhood leukaemia, polymorphisms of metabolism enzyme genes, and interactions with maternal tobacco, coffee and alcohol consumption during pregnancy." Eur J Cancer Prev **14**(6): 531-540.

259 Chiu BC, Kolar C, Gapstur SM, Lawson T, Anderson JR, et al. (2005). "Association of NAT and GST polymorphisms with non-Hodgkin's lymphoma: a population-based case-control study." Br J Haematol **128**(5): 610-615.

260 Cheng TC, Chen ST, Huang CS, Fu YP, Yu JC, et al. (2005). "Breast cancer risk associated with genotype polymorphism of the catechol estrogen-metabolizing genes: a multigenic study on cancer susceptibility." Int J Cancer **113**(3): 345-353.

261 Ceschi M, Sun CL, Van Den Berg D, Koh WP, Yu MC, et al. (2005). "The effect of cyclin D1 (CCND1) G870A-polymorphism on breast cancer risk is modified by oxidative stress among Chinese women in Singapore." Carcinogenesis **26**(8): 1457-1464.

262 Cáceres DD, Iturrieta J, Acevedo C, Huidobro C, Varela N, et al. (2005). "Relationship among metabolizing genes, smoking and alcohol used as modifier factors on prostate cancer risk: exploring some gene-gene and gene-environment interactions." Eur J Epidemiol **20**(1): 79-88.

263 Ateş NA, Tamer L, Ateş C, Ercan B, Elipek T, et al. (2005). "Glutathione S-transferase M1, T1, P1 genotypes and risk for development of colorectal cancer." Biochem Genet **43**(3-4): 149-163.

264 Yang XR, Wacholder S, Xu Z, Dean M, Clark V, et al. (2004). "CYP1A1 and GSTM1 polymorphisms in relation to lung cancer risk in Chinese women." Cancer Lett **214**(2): 197-204.

265 Xie H, Hou L, Shields PG, Winn DM, Gridley G, et al. (2004). "Metabolic polymorphisms, smoking, and oral cancer in Puerto Rico." Oncol Res **14**(6): 315-320.

266 Wrensch M, Kelsey KT, Liu M, Miike R, Moghadassi M, et al. (2004). "Glutathione-S-transferase variants and adult glioma." Cancer Epidemiol Biomarkers Prev **13**(3): 461-467.

267 Wang LI, Giovannucci EL, Hunter D, Neuberg D, Su L, et al. (2004). "Dietary intake of Cruciferous vegetables, Glutathione S-transferase (GST) polymorphisms and lung cancer risk in a Caucasian population." Cancer Causes Control **15**(10): 977-985.

268 Wang AH, Sun CS, Li LS, Huang JY, Chen QS, et al. (2004). "Genetic susceptibility and environmental factors of esophageal cancer in Xi'an." World J Gastroenterol **10**(7): 940-944

269 van der Logt EM, Bergevoet SM, Roelofs HM, van Hooijdonk Z, te Morsche RH, et al. (2004). "Genetic polymorphisms in UDP-glucuronosyltransferases and glutathione S-transferases and colorectal cancer risk." Carcinogenesis **25**(12): 2407-2415.

270 Unal M, Tamer L, Ateş NA, Akbaş Y, Pata YS, et al. (2004). "Glutathione S-transferase M1, T1, and P1 gene polymorphism in laryngeal squamous cell carcinoma." Am J Otolaryngol **25**(5): 318-322.

271 Tsukino H, Nakao H, Kuroda Y, Imai H, Inatomi H, et al. (2004). "Glutathione S-transferase (GST) M1, T1 and N-acetyltransferase 2 (NAT2) polymorphisms and urothelial cancer risk with tobacco smoking." Eur J Cancer Prev **13**(6): 509-514.

272 Tsabouri S, Georgiou I, Katsaraki A, Bourantas KL. (2004). "Glutathione sulfur transferase M1 and T1 genotypes in chronic lymphoblastic leukemia." Hematol J **5**(6): 500-504.

273 Srivastava DS, Kumar A, Mittal B, Mittal RD. (2004). "Polymorphism of GSTM1 and GSTT1 genes in bladder cancer: a study from North India." Arch Toxicol **78**(8): 430-434.

274 Sørensen M, Autrup H, Tjønneland A, Overvad K, Raaschou-Nielsen O. (2004). "Glutathione S-transferase T1 null-genotype is associated with an increased risk of lung cancer." Int J Cancer **110**(2): 219-224.

275 Sobti RC, Sharma S, Joshi A, Jindal SK, Janmeja A. (2004). "Genetic polymorphism of the CYP1A1, CYP2E1, GSTM1 and GSTT1 genes and lung cancer susceptibility in a north indian population." Mol Cell Biochem **266**(1-2): 1-9.

276 Sharma A, Sharma JK, Murthy NS, Mitra AB. (2004). "Polymorphisms at GSTM1 and GSTT1 gene loci and susceptibility to cervical cancer in Indian population." Neoplasma **51**(1): 12-16.

277 Schneider J, Bernges U, Philipp M, Woitowitz HJ. (2004). "GSTM1, GSTT1, and GSTP1 polymorphism and lung cancer risk in relation to tobacco smoking." Cancer Lett **208**(1): 65-74.

278 Sarmanová J, Sůsová S, Gut I, Mrhalová M, Kodet R, et al. (2004). "Breast cancer: role of polymorphisms in biotransformation enzymes." Eur J Hum Genet **12**(10): 848-854.

279 Sanyal S, Festa F, Sakano S, Zhang Z, Steineck G, et al. (2004). "Polymorphisms in DNA repair and metabolic genes in bladder cancer." Carcinogenesis **25**(5): 729-734.

280 Roth MJ, Abnet CC, Johnson LL, Mark SD, Dong ZW, et al. (2004). "Polymorphic variation of Cyp1A1 is associated with the risk of gastric cardia cancer: a prospective case-cohort study of cytochrome P-450 1A1 and GST enzymes." Cancer Causes Control **15**(10): 1077-1083.

281 Moore LE, Wiencke JK, Bates MN, Zheng S, Rey OA, et al. (2004). "Investigation of genetic polymorphisms and smoking in a bladder cancer case-control study in Argentina." Cancer Lett **211**(2): 199-207.

282 Mittal RD, Srivastava DS, Mandhani A, Kumar A, Mittal B. (2004). "Polymorphism of GSTM1 and GSTT1 genes in prostate cancer: a study from North India." Indian J Cancer **41**(3): 115-119.

283 Medeiros R, Vasconcelos A, Costa S, Pinto D, Ferreira P, et al. (2004). "Metabolic susceptibility genes and prostate cancer risk in a southern European population: the role of glutathione S-transferases GSTM1, GSTM3, and GSTT1 genetic polymorphisms." Prostate **58**(4): 414-420.

284 McCready D, Aronson KJ, Chu W, Fan W, Vesprini D, et al. (2004). "Breast tissue organochlorine levels and metabolic genotypes in relation to breast cancer risk Canada." Cancer Causes Control **15**(4): 399-418.

285 Lee SA, Kim JW, Roh JW, Choi JY, Lee KM, et al. (2004). "Genetic polymorphisms of GSTM1, p21, p53 and HPV infection with cervical cancer in Korean women." Gynecol Oncol **93**(1): 14-18.

286 Kiss I, Németh A, Bogner B, Pajkos G, Orsós Z, et al. (2004). "Polymorphisms of glutathione-S-transferase and arylamine N-acetyltransferase enzymes and susceptibility to colorectal cancer." Anticancer Res **24**(6): 3965-3970.

287 Joseph T, Kusumakumary P, Chacko P, Abraham A, Radhakrishna Pillai M. (2004). "Genetic polymorphism of CYP1A1, CYP2D6, GSTM1 and GSTT1 and susceptibility to acute lymphoblastic leukaemia in Indian children." Pediatr Blood Cancer **43**(5): 560-567.

288 Joseph MA, Moysich KB, Freudenheim JL, Shields PG, Bowman ED, et al. (2004). "Cruciferous vegetables, genetic polymorphisms in glutathione S-transferases M1 and T1, and prostate cancer risk." Nutr Cancer **50**(2): 206-213.

289 Hung RJ, Boffetta P, Brennan P, Malaveille C, Hautefeuille A, et al. (2004). "GST, NAT, SULT1A1, CYP1B1 genetic polymorphisms, interactions with environmental exposures and bladder cancer risk in a high-risk population." Int J Cancer **110**(4): 598-604.

290 Harms C, Salama SA, Sierra-Torres CH, Cajas-Salazar N, Au WW. et al. (2004). "Polymorphisms in DNA repair genes, chromosome aberrations, and lung cancer." Environ Mol Mutagen **44**(1): 74-82.

291 Habalová V, Salagovic J, Kalina I, Stubna J. (2004). "Combined analysis of polymorphisms in glutathione S-transferase M1 and microsomal epoxide hydrolase in lung cancer patients." Neoplasma **51**(5): 352-357.

292 Gaudet MM, Olshan AF, Poole C, Weissler MC, Watson M， et al. (2004). "Diet, GSTM1 and GSTT1 and head and neck cancer." Carcinogenesis **25**(5): 735-740.

293 Gaspar J, Rodrigues S, Gil OM, Manita I, Ferreira TC, et al. (2004). "Combined effects of glutathione S-transferase polymorphisms and thyroid cancer risk." Cancer Genet Cytogenet **151**(1): 60-67.

294 Gago-Dominguez M, Castelao JE, Sun CL, Van Den Berg D, Koh WP, et al. (2004). "Marine n-3 fatty acid intake, glutathione S-transferase polymorphisms and breast cancer risk in post-menopausal Chinese women in Singapore." Carcinogenesis **25**(11): 2143-2147.

295 Evans AJ, Henner WD, Eilers KM, Montalto MA, Wersinger EM, et al. (2004). "Polymorphisms of GSTT1 and related genes in head and neck cancer risk." Head Neck **26**(1): 63-70.

296 Egan KM, Cai Q, Shu XO, Jin F, Zhu TL, et al. (2004). "Genetic polymorphisms in GSTM1, GSTP1, and GSTT1 and the risk for breast cancer: results from the Shanghai Breast Cancer Study and meta-analysis." Cancer Epidemiol Biomarkers Prev **13**(2): 197-204.

297 Drummond SN, De Marco L, Noronha JC, Gomez RS. (2004). "GSTM1 polymorphism and oral squamous cell carcinoma." Oral Oncol **40**(1): 52-55.

298 D'Alò F, Voso MT, Guidi F, Massini G, Scardocci A, et al. (2004). "Polymorphisms of CYP1A1 and glutathione S-transferase and susceptibility to adult acute myeloid leukemia." Haematologica **89**(6): 664-670.

299 Colombo J, Rossit AR, Caetano A, Borim AA, Wornrath D, et al. (2004). "GSTT1, GSTM1 and CYP2E1 genetic polymorphisms in gastric cancer and chronic gastritis in a Brazilian population." World J Gastroenterol **10**(9): 1240-1245.

300 Chen YC, Xu L, Guo YL, Su HJ, Smith TJ, et al. (2004). "Polymorphisms in GSTT1 and p53 and urinary transitional cell carcinoma in south-western Taiwan: a preliminary study." Biomarkers **9**(4-5): 386-394.

301 C Chan-Yeung M, Tan-Un KC, Ip MS, Tsang KW, Ho SP,han-Yeung, M., K. C. Tan-Un, et al. (2004). "Lung cancer susceptibility and polymorphisms of glutathione-S-transferase genes in Hong Kong." Lung Cancer **45**(2): 155-160.

302 Canalle R, Burim RV, Tone LG, Takahashi CS. (2004). "Genetic polymorphisms and susceptibility to childhood acute lymphoblastic leukemia." Environ Mol Mutagen **43**(2): 100-109.

303 Belogubova EV, Togo AV, Karpova MB, Kuligina ESh, Buslova KG, et al. (2004). "A novel approach for assessment of cancer predisposing roles of GSTM1 and GSTT1 genes: use of putatively cancer resistant elderly tumor-free smokers as the referents." Lung Cancer **43**(3): 259-266.

304 Alexandrie AK, Nyberg F, Warholm M, Rannug A. (2004). "Influence of CYP1A1, GSTM1, GSTT1, and NQO1 genotypes and cumulative smoking dose on lung cancer risk in a Swedish population." Cancer Epidemiol Biomarkers Prev **13**(6): 908-914.

305 Aktas D, Hascicek M, Sozen S, Ozen H, Tuncbilek E. (2004). "CYP1A1 and GSTM1 polymorphic genotypes in patients with prostate cancer in a Turkish population." Cancer Genet Cytogenet **154**(1): 81-85.

306 Abbas A, Delvinquiere K, Lechevrel M, Lebailly P, Gauduchon P, et al. (2004). "GSTM1, GSTT1, GSTP1 and CYP1A1 genetic polymorphisms and susceptibility to esophageal cancer in a French population: different pattern of squamous cell carcinoma and adenocarcinoma." World J Gastroenterol **10**(23): 3389-3393.

307 Wang LD, Zheng S, Liu B, Zhou JX, Li YJ, et al. (2003). "CYP1A1, GSTs and mEH polymorphisms and susceptibility to esophageal carcinoma: study of population from a high- incidence area in north China." World J Gastroenterol **9**(7): 1394-1397.

308 Wang J, Deng Y, Li L, Kuriki K, Ding J, et al. (2003). "Association of GSTM1, CYP1A1 and CYP2E1 genetic polymorphisms with susceptibility to lung adenocarcinoma: a case-control study in Chinese population." Cancer Sci **94**(5): 448-452.

309 van der Hel OL, Peeters PH, Hein DW, Doll MA, Grobbee DE et al. (2003). "NAT2 slow acetylation and GSTM1 null genotypes may increase postmenopausal breast cancer risk in long-term smoking women." Pharmacogenetics **13**(7): 399-407.

310 van der Hel OL, Bueno de Mesquita HB, Roest M, Slothouber B, van Gils C,et al. (2003). "No modifying effect of NAT1, GSTM1, and GSTT1 on the relation between smoking and colorectal cancer risk." Cancer Epidemiol Biomarkers Prev **12**(7): 681-682.

311 Tsai YY, McGlynn KA, Hu Y, Cassidy AB, Arnold J, et al. (2003). "Genetic susceptibility and dietary patterns in lung cancer." Lung Cancer **41**(3): 269-281.

312 Taioli E, Gaspari L, Benhamou S, Boffetta P, Brockmoller J, et al. (2003). "Polymorphisms in CYP1A1, GSTM1, GSTT1 and lung cancer below the age of 45 years." Int J Epidemiol **32**(1): 60-63.

313 Slattery ML, Edwards S, Curtin K, Schaffer D, Neuhausen S. (2003). "Associations between smoking, passive smoking, GSTM-1, NAT2, and rectal cancer." Cancer Epidemiol Biomarkers Prev **12**(9): 882-889.

314 Sierra-Torres CH, Au WW, Arrastia CD, Cajas-Salazar N, Robazetti SC, et al. (2003). "Polymorphisms for chemical metabolizing genes and risk for cervical neoplasia." Environ Mol Mutagen **41**(1): 69-76.

315 Schroeder JC, Conway K, Li Y, Mistry K, Bell DA, et al. (2003). "p53 mutations in bladder cancer: evidence for exogenous versus endogenous risk factors." Cancer Res **63**(21): 7530-7538.

316 Ruano-Ravina A, Figueiras A, Loidi L, Barros-Dios JM. (2003). "GSTM1 and GSTT1 polymorphisms, tobacco and risk of lung cancer: a case-control study from Galicia, Spain." Anticancer Res **23**(5b): 4333-4337.

317 Risch A, Ramroth H, Raedts V, Rajaee-Behbahani N, Schmezer P, et al. (2003). "Laryngeal cancer risk in Caucasians is associated with alcohol and tobacco consumption but not modified by genetic polymorphisms in class I alcohol dehydrogenases ADH1B and ADH1C, and glutathione-S-transferases GSTM1 and GSTT1." Pharmacogenetics **13**(4): 225-230.

318 Reszka E, Wasowicz W, Rydzynski K, Szeszenia-Dabrowska N, Szymczak W. (2003). "Glutathione S-transferase M1 and P1 metabolic polymorphism and lung cancer predisposition." Neoplasma **50**(5): 357-362.

319 Pinarbasi H, Silig Y, Cetinkaya O, Seyfikli Z, Pinarbasi E. (2003). "Strong association between the GSTM1-null genotype and lung cancer in a Turkish population." Cancer Genet Cytogenet **146**(2): 125-129.

320 Park SK, Kang D, Noh DY, Lee KM, Kim SU, et al. (2003). "Reproductive factors, glutathione S-transferase M1 and T1 genetic polymorphism and breast cancer risk." Breast Cancer Res Treat **78**(1): 89-96.

321 Nazar-Stewart V, Vaughan TL, Stapleton P, Van Loo J, Nicol-Blades B, et al. (2003). "A population-based study of glutathione S-transferase M1, T1 and P1 genotypes and risk for lung cancer." Lung Cancer **40**(3): 247-258.

322 Nakazato H, Suzuki K, Matsui H, Koike H, Okugi H,et al. (2003). "Association of genetic polymorphisms of glutathione-S-transferase genes (GSTM1, GSTT1 and GSTP1) with familial prostate cancer risk in a Japanese population." Anticancer Res **23**(3C): 2897-2902.

323 Munaka M, Kohshi K, Kawamoto T, Takasawa S, Nagata N, et al. (2003). "Genetic polymorphisms of tobacco- and alcohol-related metabolizing enzymes and the risk of hepatocellular carcinoma." J Cancer Res Clin Oncol **129**(6): 355-360.

324 Mathonnet G, Krajinovic M, Labuda D, Sinnett D. (2003). "Role of DNA mismatch repair genetic polymorphisms in the risk of childhood acute lymphoblastic leukaemia." Br J Haematol **123**(1): 45-48.

325 Kiyohara C, Wakai K, Mikami H, Sido K, Ando M, et al. (2003). "Risk modification by CYP1A1 and GSTM1 polymorphisms in the association of environmental tobacco smoke and lung cancer: a case-control study in Japanese nonsmoking women." Int J Cancer **107**(1): 139-144.

326 Khedhaier A, Remadi S, Corbex M, Ahmed SB, Bouaouina N, et al. (2003). "Glutathione S-transferases (GSTT1 and GSTM1) gene deletions in Tunisians: susceptibility and prognostic implications in breast carcinoma." Br J Cancer **89**(8): 1502-1507.

327 Kang, D. (2003). "Genetic polymorphisms and cancer susceptibility of breast cancer in Korean women." J Biochem Mol Biol **36**(1): 28-34.

328 Jong Jeong H, Jin Kim H, Young Seo I, Ju Kim H, Oh GJ, et al. (2003). "Association between glutathione S-transferase M1 and T1 polymorphisms and increased risk for bladder cancer in Korean smokers." Cancer Lett **202**(2): 193-199.

329 Hohaus S, Massini G, D'Alo' F, Guidi F, Putzulu R, et al. (2003). "Association between glutathione S-transferase genotypes and Hodgkin's lymphoma risk and prognosis." Clin Cancer Res **9**(9): 3435-3440.

330 Hernández A, Céspedes W, Xamena N, Surrallés J, Creus A, et al. (2003). "Glutathione S-transferase polymorphisms in thyroid cancer patients." Cancer Lett **190**(1): 37-44.

331 Gronau S, Koenig-Greger D, Jerg M, Riechelmann H. (2003). "GSTM1 enzyme concentration and enzyme activity in correlation to the genotype of detoxification enzymes in squamous cell carcinoma of the oral cavity." Oral Dis **9**(2): 62-67.

332 Gallegos-Arreola MP, Gómez-Meda BC, Morgan-Villela G, Arechavaleta-Granell MR, Arnaud-López L, et al. (2003). "GSTT1 gene deletion is associated with lung cancer in Mexican patients." Dis Markers **19**(6): 259-261.

334 De Roos AJ, Rothman N, Inskip PD, Linet MS, Shapiro WR, et al. (2003). "Genetic polymorphisms in GSTM1, -P1, -T1, and CYP2E1 and the risk of adult brain tumors." Cancer Epidemiol Biomarkers Prev **12**(1): 14-22.

335 Cheng YJ, Chien YC, Hildesheim A, Hsu MM, Chen IH, et al. (2003). "No association between genetic polymorphisms of CYP1A1, GSTM1, GSTT1, GSTP1, NAT2, and nasopharyngeal carcinoma in Taiwan." Cancer Epidemiol Biomarkers Prev **12**(2): 179-180

336 Casson AG, Zheng Z, Chiasson D, MacDonald K, Riddell DC, et al. (2003). "Associations between genetic polymorphisms of Phase I and II metabolizing enzymes, p53 and susceptibility to esophageal adenocarcinoma." Cancer Detect Prev **27**(2): 139-146.

337 Canbay E, Dokmetas S, Canbay EI, Sen M, Bardakci F. (2003). "Higher glutathione transferase GSTM1 0/0 genotype frequency in young thyroid carcinoma patients." Curr Med Res Opin **19**(2): 102-106.

338 Cajas-Salazar N, Sierra-Torres CH, Salama SA, Zwischenberger JB, Au WW. (2003). "Combined effect of MPO, GSTM1 and GSTT1 polymorphisms on chromosome aberrations and lung cancer risk." Int J Hyg Environ Health **206**(6): 473-483.

339 Buzio L, De Palma G, Mozzoni P, Tondel M, Buzio C, et al. (2003). "Glutathione S-transferases M1-1 and T1-1 as risk modifiers for renal cell cancer associated with occupational exposure to chemicals." Occup Environ Med **60**(10): 789-793.

340 Bardakci F, Canbay E, Degerli N, Coban L, Canbay EI. (2003). "Relationship of tobacco smoking with GSTM1 gene polymorphism in laringeal cancer." J Cell Mol Med **7**(3): 307-312.

341 Acevedo C, Opazo JL, Huidobro C, Cabezas J, Iturrieta J, et al. (2003). "Positive correlation between single or combined genotypes of CYP1A1 and GSTM1 in relation to prostate cancer in Chilean people." Prostate **57**(2): 111-117.

342 Zheng W, Wen WQ, Gustafson DR, Gross M, Cerhan JR, et al. (2002). "GSTM1 and GSTT1 polymorphisms and postmenopausal breast cancer risk." Breast Cancer Res Treat **74**(1): 9-16.

343 Zheng T, Holford TR, Zahm SH, Owens PH, Boyle P, et al. (2002). "Cigarette smoking, glutathione-s-transferase M1 and t1 genetic polymorphisms, and breast cancer risk (United States)." Cancer Causes Control **13**(7): 637-645.

344 Yuille M, Condie A, Hudson C, Kote-Jarai Z, Stone E, et al. (2002). "Relationship between glutathione S-transferase M1, T1, and P1 polymorphisms and chronic lymphocytic leukemia." Blood **99**(11): 4216-4218.

345 Yokoyama A, Kato H, Yokoyama T, Tsujinaka T, Muto M, et al. (2002). "Genetic polymorphisms of alcohol and aldehyde dehydrogenases and glutathione S-transferase M1 and drinking, smoking, and diet in Japanese men with esophageal squamous cell carcinoma." Carcinogenesis **23**(11): 1851-1859.

346 Ye, Z. and J. M. Parry (2002). "Genetic polymorphisms in the cytochrome P450 1A1, glutathione S-transferase M1 and T1, and susceptibility to colon cancer." Teratog Carcinog Mutagen **22**(5): 385-392.

347 Wu MS, Chen CJ, Lin MT, Wang HP, Shun CT, et al. (2002). "Genetic polymorphisms of cytochrome p450 2E1, glutathione S-transferase M1 and T1, and susceptibility to gastric carcinoma in Taiwan." Int J Colorectal Dis **17**(5): 338-343.

348 Wu FY, Lee YJ, Chen DR, Kuo HW. (2002). "Association of DNA-protein crosslinks and breast cancer." Mutat Res **501**(1-2): 69-78.

350 To-Figueras J, Gené M, Gómez-Catalán J, Piqué E, Borrego N, et al. (2002). "Microsomal epoxide hydrolase and glutathione S-transferase polymorphisms in relation to laryngeal carcinoma risk." Cancer Lett **187**(1-2): 95-101.

351 Tiemersma EW, Kampman E, Bueno de Mesquita HB, Bunschoten A, van Schothorst EM, et al. (2002). "Meat consumption, cigarette smoking, and genetic susceptibility in the etiology of colorectal cancer: results from a Dutch prospective study." Cancer Causes Control **13**(4): 383-393.

352 Sunaga N, Kohno T, Yanagitani N, Sugimura H, Kunitoh H, et al. (2002). "Contribution of the NQO1 and GSTT1 polymorphisms to lung adenocarcinoma susceptibility." Cancer Epidemiol Biomarkers Prev **11**(8): 730-738.

353 Stücker I, Hirvonen A, de Waziers I, Cabelguenne A, Mitrunen K, et al. (2002). "Genetic polymorphisms of glutathione S-transferases as modulators of lung cancer susceptibility." Carcinogenesis **23**(9): 1475-1481.

354 Siegelmann-Danieli, N. and K. H. Buetow (2002). "Significance of genetic variation at the glutathione S-transferase M1 and NAD(P)H:quinone oxidoreductase 1 detoxification genes in breast cancer development." Oncology **62**(1): 39-45.

355 Seow A, Yuan JM, Sun CL, Van Den Berg D, Lee HP, et al. (2002). "Dietary isothiocyanates, glutathione S-transferase polymorphisms and colorectal cancer risk in the Singapore Chinese Health Study." Carcinogenesis **23**(12): 2055-2061.

356 Perera FP, Mooney LA, Stampfer M, Phillips DH, Bell DA, et al. (2002). "Associations between carcinogen-DNA damage, glutathione S-transferase genotypes, and risk of lung cancer in the prospective Physicians' Health Cohort Study." Carcinogenesis **23**(10): 1641-1646.

357 Miller DP, Liu G, De Vivo I, Lynch TJ, Wain JC, et al. (2002). "Combinations of the variant genotypes of GSTP1, GSTM1, and p53 are associated with an increased lung cancer risk." Cancer Res **62**(10): 2819-2823.

358 Matheson MC, Stevenson T, Akbarzadeh S, Propert DN. (2002). "GSTT1 null genotype increases risk of premenopausal breast cancer." Cancer Lett **181**(1): 73-79.

359 Lu W, Xing D, Qi J, Tan W, Miao X, et al. (2002). "Genetic polymorphism in myeloperoxidase but not GSTM1 is associated with risk of lung squamous cell carcinoma in a Chinese population." Int J Cancer **102**(3): 275-279.

360 Lewis SJ, Cherry NM, Niven RM, Barber PV, Povey AC. (2002). "GSTM1, GSTT1 and GSTP1 polymorphisms and lung cancer risk." Cancer Lett **180**(2): 165-171.

361 Lee SJ, Cho SH, Park SK, Kim SW, Park MS,et al. (2002). "Combined effect of glutathione S-transferase M1 and T1 genotypes on bladder cancer risk." Cancer Lett **177**(2): 173-179.

362 Kim WJ, Kim H, Kim CH, Lee MS, Oh BR, et al. (2002). "GSTT1-null genotype is a protective factor against bladder cancer." Urology **60**(5): 913-918.

363 Kerridge I, Lincz L, Scorgie F, Hickey D, Granter N, et al. (2002). "Association between xenobiotic gene polymorphisms and non-Hodgkin's lymphoma risk." Br J Haematol **118**(2): 477-481.

364 Hahn M, Hagedorn G, Kuhlisch E, Schackert HK, Eckelt U. (2002). "Genetic polymorphisms of drug-metabolizing enzymes and susceptibility to oral cavity cancer." Oral Oncol **38**(5): 486-490.

365 Giannakopoulos X, Charalabopoulos K, Baltogiannis D, Chatzikiriakidou A, Alamanos Y, et al. (2002). "The role of N-acetyltransferase-2 and glutathione S-transferase on the risk and aggressiveness of bladder cancer." Anticancer Res **22**(6B): 3801-3804.

366 Gao CM, Takezaki T, Wu JZ, Li ZY, Liu YT,et al. (2002). "Glutathione-S-transferases M1 (GSTM1) and GSTT1 genotype, smoking, consumption of alcohol and tea and risk of esophageal and stomach cancers: a case-control study of a high-incidence area in Jiangsu Province, China." Cancer Lett **188**(1-2): 95-102.

367 da Fonte de Amorim L, Rossini A, Mendonça G, Lotsch P, de Almeida Simão T, d et al. (2002). "CYP1A1, GSTM1, and GSTT1 polymorphisms and breast cancer risk in Brazilian women." Cancer Lett **181**(2): 179-186.

368 Buch SC, Notani PN, Bhisey RA. (2002). "Polymorphism at GSTM1, GSTM3 and GSTT1 gene loci and susceptibility to oral cancer in an Indian population." Carcinogenesis **23**(5): 803-807.

369 Beer TM, Evans AJ, Hough KM, Lowe BA, McWilliams JE, et al. (2002). "Polymorphisms of GSTP1 and related genes and prostate cancer risk." Prostate Cancer Prostatic Dis **5**(1): 22-27.

370 Alves S, Amorim A, Ferreira F, Norton L, Prata MJ.. (2002). "The GSTM1 and GSTT1 genetic polymorphisms and susceptibility to acute lymphoblastic leukemia in children from north Portugal." Leukemia **16**(8): 1565-1567.

371 Zhao B, Seow A, Lee EJ, Poh WT, Teh M, et al. (2001). "Dietary isothiocyanates, glutathione S-transferase -M1, -T1 polymorphisms and lung cancer risk among Chinese women in Singapore." Cancer Epidemiol Biomarkers Prev **10**(10): 1063-1067.

372 Xiong P, Bondy ML, Li D, Shen H, Wang LE, et al. (2001). "Sensitivity to benzo(a)pyrene diol-epoxide associated with risk of breast cancer in young women and modulation by glutathione S-transferase polymorphisms: a case-control study." Cancer Res **61**(23): 8465-8469.

373 Törüner GA, Akyerli C, Uçar A, Aki T, Atsu N, et al. (2001). "Polymorphisms of glutathione S-transferase genes (GSTM1, GSTP1 and GSTT1) and bladder cancer susceptibility in the Turkish population." Arch Toxicol **75**(8): 459-464.

374 To-Figueras J, Gené M, Gómez-Catalán J, Piqué E, Borrego N, et al. (2001). "Lung cancer susceptibility in relation to combined polymorphisms of microsomal epoxide hydrolase and glutathione S-transferase P1." Cancer Lett **173**(2): 155-162.

375 Tiemersma EW, Omer RE, Bunschoten A, van't Veer P, Kok FJ, et al. (2001). "Role of genetic polymorphism of glutathione-S-transferase T1 and microsomal epoxide hydrolase in aflatoxin-associated hepatocellular carcinoma." Cancer Epidemiol Biomarkers Prev **10**(7): 785-791.

376 Sun CA, Wang LY, Chen CJ, Lu SN, You SL, et al. (2001). "Genetic polymorphisms of glutathione S-transferases M1 and T1 associated with susceptibility to aflatoxin-related hepatocarcinogenesis among chronic hepatitis B carriers: a nested case-control study in Taiwan." Carcinogenesis **22**(8): 1289-1294.

377 Sreelekha TT, Ramadas K, Pandey M, Thomas G, Nalinakumari KR, et al. (2001). "Genetic polymorphism of CYP1A1, GSTM1 and GSTT1 genes in Indian oral cancer." Oral Oncol **37**(7): 593-598.

378 Spurdle AB, Webb PM, Purdie DM, Chen X, Green A, et al. (2001). "Polymorphisms at the glutathione S-transferase GSTM1, GSTT1 and GSTP1 loci: risk of ovarian cancer by histological subtype." Carcinogenesis **22**(1): 67-72.

379 Saadat, I. and M. Saadat (2001). "Glutathione S-transferase M1 and T1 null genotypes and the risk of gastric and colorectal cancers." Cancer Lett **169**(1): 21-26.

380 Risch A, Wikman H, Thiel S, Schmezer P, Edler L,et al. (2001). "Glutathione-S-transferase M1, M3, T1 and P1 polymorphisms and susceptibility to non-small-cell lung cancer subtypes and hamartomas." Pharmacogenetics **11**(9): 757-764.

381 Quiñones L, Lucas D, Godoy J, Cáceres D, Berthou F, et al. (2001). "CYP1A1, CYP2E1 and GSTM1 genetic polymorphisms. The effect of single and combined genotypes on lung cancer susceptibility in Chilean people." Cancer Lett **174**(1): 35-44.

382 Murata M, Watanabe M, Yamanaka M, Kubota Y, Ito H, et al. (2001). "Genetic polymorphisms in cytochrome P450 (CYP) 1A1, CYP1A2, CYP2E1, glutathione S-transferase (GST) M1 and GSTT1 and susceptibility to prostate cancer in the Japanese population." Cancer Lett **165**(2): 171-177.

383 Mitrunen K, Jourenkova N, Kataja V, Eskelinen M, Kosma VM, et al. (2001). "Glutathione S-transferase M1, M3, P1, and T1 genetic polymorphisms and susceptibility to breast cancer." Cancer Epidemiol Biomarkers Prev **10**(3): 229-236.

384 Maugard CM, Charrier J, Pitard A, Campion L, Akande O, et al. (2001). "Genetic polymorphism at the glutathione S-transferase (GST) P1 locus is a breast cancer risk modifier." Int J Cancer **91**(3): 334-339.

385 Loktionov A, Watson MA, Gunter M, Stebbings WS, Speakman CT,et al. (2001). "Glutathione-S-transferase gene polymorphisms in colorectal cancer patients: interaction between GSTM1 and GSTM3 allele variants as a risk-modulating factor." Carcinogenesis **22**(7): 1053-1060.

386 Lewis S, Brennan P, Nyberg F, Ahrens W, Constantinescu V, et al. (2001). "Re: Spitz, M. R., Duphorne, C. M., Detry, M. A., Pillow, P. C., Amos, C. I., Lei, L., de Andrade, M., Gu, X., Hong, W. K., and Wu, X. Dietary intake of isothiocyanates: evidence of a joint effect with glutathione S-transferase polymorphisms in lung cancer risk. Cancer Epidemiol. Biomark. Prev., 9: 1017-1020, 2000." Cancer Epidemiol Biomarkers Prev **10**(10): 1105-1106.

387 Krajinovic M, Ghadirian P, Richer C, Sinnett H, Gandini S, et al. (2001). "Genetic susceptibility to breast cancer in French-Canadians: role of carcinogen-metabolizing enzymes and gene-environment interactions." Int J Cancer **92**(2): 220-225.

388 Kote-Jarai Z, Easton D, Edwards SM, Jefferies S, Durocher F,et al. (2001). "Relationship between glutathione S-transferase M1, P1 and T1 polymorphisms and early onset prostate cancer." Pharmacogenetics **11**(4): 325-330.

388 Kietthubthew S, Sriplung H, Au WW. (2001). "Genetic and environmental interactions on oral cancer in Southern Thailand." Environ Mol Mutagen **37**(2): 111-116.

389 Hou SM, Fält S, Yang K, Nyberg F, Pershagen G, et al. (2001). "Differential interactions between GSTM1 and NAT2 genotypes on aromatic DNA adduct level and HPRT mutant frequency in lung cancer patients and population controls." Cancer Epidemiol Biomarkers Prev **10**(2): 133-140

390 Gudmundsdottir K, Tryggvadottir L, Eyfjord JE. (2001). "GSTM1, GSTT1, and GSTP1 genotypes in relation to breast cancer risk and frequency of mutations in the p53 gene." Cancer Epidemiol Biomarkers Prev **10**(11): 1169-1173.

391 Gsur A, Haidinger G, Hollaus P, Herbacek I, Madersbacher S, et al. (2001). "Genetic polymorphisms of CYP1A1 and GSTM1 and lung cancer risk." Anticancer Res **21**(3C): 2237-2242.

393 Chen S, Xue K, Xu L, Ma G, Wu J. (2001). "Polymorphisms of the CYP1A1 and GSTM1 genes in relation to individual susceptibility to lung carcinoma in Chinese population." Mutat Res **458**(1-2): 41-47.

394 Cai L, Yu SZ, Zhang ZF. (2001). "Glutathione S-transferases M1, T1 genotypes and the risk of gastric cancer: a case-control study." World J Gastroenterol **7**(4): 506-509.

395 Butler WJ, Ryan P, Roberts-Thomson IC. (2001). "Metabolic genotypes and risk for colorectal cancer." J Gastroenterol Hepatol **16**(6): 631-635.

396 Baxter SW, Thomas EJ, Campbell IG. (2001). "GSTM1 null polymorphism and susceptibility to endometriosis and ovarian cancer." Carcinogenesis **22**(1): 63-65.

397 Aktas D, Ozen H, Atsu N, Tekin A, Sozen S, et al. (2001). "Glutathione S-transferase M1 gene polymorphism in bladder cancer patients. a marker for invasive bladder cancer?" Cancer Genet Cytogenet **125**(1): 1-4.

398 Tan W, Song N, Wang GQ, Liu Q, Tang HJ, et al. (2000). "Impact of genetic polymorphisms in cytochrome P450 2E1 and glutathione S-transferases M1, T1, and P1 on susceptibility to esophageal cancer among high-risk individuals in China." Cancer Epidemiol Biomarkers Prev **9**(6): 551-556.

399 Sweeney C, Farrow DC, Schwartz SM, Eaton DL, Checkoway H, et al. (2000). "Glutathione S-transferase M1, T1, and P1 polymorphisms as risk factors for renal cell carcinoma: a case-control study." Cancer Epidemiol Biomarkers Prev **9**(4): 449-454.

400 Stücker I, Jacquet M, de Waziers I, Cénée S, Beaune P,et al. (2000). "Relation between inducibility of CYP1A1, GSTM1 and lung cancer in a French population." Pharmacogenetics **10**(7): 617-627.

401 Steinhoff C, Franke KH, Golka K, Thier R, Römer HC, et al. (2000). "Glutathione transferase isozyme genotypes in patients with prostate and bladder carcinoma." Arch Toxicol **74**(9): 521-526.

402 Stanulla M, Schrappe M, Brechlin AM, Zimmermann M, Welte K. (2000). "Polymorphisms within glutathione S-transferase genes (GSTM1, GSTT1, GSTP1) and risk of relapse in childhood B-cell precursor acute lymphoblastic leukemia: a case-control study." Blood **95**(4): 1222-1228.

403 Setiawan VW, Zhang ZF, Yu GP, Li YL, Lu ML, et al. (2000). "GSTT1 and GSTM1 null genotypes and the risk of gastric cancer: a case-control study in a Chinese population." Cancer Epidemiol Biomarkers Prev **9**(1): 73-80.

404 Schnakenberg E, Lustig M, Breuer R, Werdin R, Hübotter R, et al. (2000). "Gender-specific effects of NAT2 and GSTM1 in bladder cancer." Clin Genet **57**(4): 270-277.

405 Sato M, Sato T, Izumo T, Amagasa T. (2000). "Genetically high susceptibility to oral squamous cell carcinoma in terms of combined genotyping of CYP1A1 and GSTM1 genes." Oral Oncol **36**(3): 267-271.

406 Park SK, Yoo KY, Lee SJ, Kim SU, Ahn SH,et al. (2000). "Alcohol consumption, glutathione S-transferase M1 and T1 genetic polymorphisms and breast cancer risk." Pharmacogenetics **10**(4): 301-309.

407 Olshan AF, Weissler MC, Watson MA, Bell DA. (2000). "GSTM1, GSTT1, GSTP1, CYP1A1, and NAT1 polymorphisms, tobacco use, and the risk of head and neck cancer." Cancer Epidemiol Biomarkers Prev **9**(2): 185-191.

408 Mungan NA, Aben KK, Beeks E, Kampman E, Bunschoten A,et al. (2000). "A germline homozygote deletion of the glutathione-S-transferase Mu1 gene predisposes to bladder cancer." Urol Int **64**(3): 134-138.

409 McWilliams JE, Evans AJ, Beer TM, Andersen PE, Cohen JI, et al. (2000). "Genetic polymorphisms in head and neck cancer risk." Head Neck **22**(6): 609-617.

410 Malats N, Camus-Radon AM, Nyberg F, Ahrens W, Constantinescu V, et al. (2000). "Lung cancer risk in nonsmokers and GSTM1 and GSTT1 genetic polymorphism." Cancer Epidemiol Biomarkers Prev **9**(8): 827-833.

411 London SJ, Yuan JM, Coetzee GA, Gao YT, Ross RK, et al. (2000). "CYP1A1 I462V genetic polymorphism and lung cancer risk in a cohort of men in Shanghai, China." Cancer Epidemiol Biomarkers Prev **9**(9): 987-991.

412 Liu G, Ghadirian P, Vesprini D, Hamel N, Paradis AJ, et al. (2000). "Polymorphisms in GSTM1, GSTT1 and CYP1A1 and risk of pancreatic adenocarcinoma." Br J Cancer **82**(10): 1646-1649.

413 Lan Q, He X, Costa DJ, Tian L, Rothman N, et al. (2000). "Indoor coal combustion emissions, GSTM1 and GSTT1 genotypes, and lung cancer risk: a case-control study in Xuan Wei, China." Cancer Epidemiol Biomarkers Prev **9**(6): 605-608.

414 Lallas TA, McClain SK, Shahin MS, Buller RE. (2000). "The glutathione S-transferase M1 genotype in ovarian cancer." Cancer Epidemiol Biomarkers Prev **9**(6): 587-590.

415 Kim WJ, Lee HL, Lee SC, Kim YT, Kim H. (2000). "Polymorphisms of N-acetyltransferase 2, glutathione S-transferase mu and theta genes as risk factors of bladder cancer in relation to asthma and tuberculosis." J Urol **164**(1): 209-213.

416 Kim JW, Lee CG, Park YG, Kim KS, Kim IK, et al. (2000). "Combined analysis of germline polymorphisms of p53, GSTM1, GSTT1, CYP1A1, and CYP2E1: relation to the incidence rate of cervical carcinoma." Cancer **88**(9): 2082-2091.

417 Goodman JE, Lavigne JA, Hengstler JG, Tanner B, Helzlsouer KJ, et al. (2000). "Catechol-O-methyltransferase polymorphism is not associated with ovarian cancer risk." Cancer Epidemiol Biomarkers Prev **9**(12): 1373-1376.

418 Ford JG, Li Y, O'Sullivan MM, Demopoulos R, Garte S,et al. (2000). "Glutathione S-transferase M1 polymorphism and lung cancer risk in African-Americans." Carcinogenesis **21**(11): 1971-1975.

419 Dresler CM, Fratelli C, Babb J, Everley L, Evans AA, et al. (2000). "Gender differences in genetic susceptibility for lung cancer." Lung Cancer **30**(3): 153-160.

420 Curran JE, Weinstein SR, Griffiths LR. (2000). "Polymorphisms of glutathione S-transferase genes (GSTM1, GSTP1 and GSTT1) and breast cancer susceptibility." Cancer Lett **153**(1-2): 113-120.

421 Crump C, Chen C, Appelbaum FR, Kopecky KJ, Schwartz SM, et al. (2000). "Glutathione S-transferase theta 1 gene deletion and risk of acute myeloid leukemia." Cancer Epidemiol Biomarkers Prev **9**(5): 457-460

422  Yoshioka M, Katoh T, Nakano M, Takasawa S, Nagata N, et al. (1999). "Glutathione S-transferase (GST) M1, T1, P1, N-acetyltransferase (NAT) 1 and 2 genetic polymorphisms and susceptibility to colorectal cancer." J UOEH **21**(2): 133-147.

423 Welfare M, Monesola Adeokun A, Bassendine MF, Daly AK. (1999). "Polymorphisms in GSTP1, GSTM1, and GSTT1 and susceptibility to colorectal cancer." Cancer Epidemiol Biomarkers Prev **8**(4 Pt 1): 289-292.

424 Tanimoto K, Hayashi S, Yoshiga K, Ichikawa T. (1999). "Polymorphisms of the CYP1A1 and GSTM1 gene involved in oral squamous cell carcinoma in association with a cigarette dose." Oral Oncol **35**(2): 191-196.

425 Stücker I, de Waziers I, Cenée S, Bignon J, Depierre A,et al. (1999). "GSTM1, smoking and lung cancer: a case-control study." Int J Epidemiol **28**(5): 829-835.

426 Sato M, Sato T, Izumo T, Amagasa T. (1999). "Genetic polymorphism of drug-metabolizing enzymes and susceptibility to oral cancer." Carcinogenesis **20**(10): 1927-1931.

427 Salagovic J, Kalina I, Habalová V, Hrivnák M, Valanský L, et al. (1999). "The role of human glutathione S-transferases M1 and T1 in individual susceptibility to bladder cancer." Physiol Res **48**(6): 465-471.

428 Rebbeck TR, Walker AH, Jaffe JM, White DL, Wein AJ,et al. (1999). "Glutathione S-transferase-mu (GSTM1) and -theta (GSTT1) genotypes in the etiology of prostate cancer." Cancer Epidemiol Biomarkers Prev **8**(4 Pt 1): 283-287.

429 Nazar-Stewart V, Vaughan TL, Burt RD, Chen C, Berwick M, et al. (1999). "Glutathione S-transferase M1 and susceptibility to nasopharyngeal carcinoma." Cancer Epidemiol Biomarkers Prev **8**(6): 547-551.

430 Morita S, Yano M, Tsujinaka T, Akiyama Y, Taniguchi M,et al. (1999). "Genetic polymorphisms of drug-metabolizing enzymes and susceptibility to head-and-neck squamous-cell carcinoma." Int J Cancer **80**(5): 685-688.

431 Longuemaux S, Deloménie C, Gallou C, Méjean A, Vincent-Viry M, et al. (1999). "Candidate genetic modifiers of individual susceptibility to renal cell carcinoma: a study of polymorphic human xenobiotic-metabolizing enzymes." Cancer Res **59**(12): 2903-2908.

432 Krajinovic M, Labuda D, Richer C, Karimi S, Sinnett D. (1999). "Susceptibility to childhood acute lymphoblastic leukemia: influence of CYP1A1, CYP2D6, GSTM1, and GSTT1 genetic polymorphisms." Blood **93**(5): 1496-1501.

433 Katoh T, Kaneko S, Kohshi K, Munaka M, Kitagawa K, et al. (1999). "Genetic polymorphisms of tobacco- and alcohol-related metabolizing enzymes and oral cavity cancer." Int J Cancer **83**(5): 606-609.

434 Kampman E, Slattery ML, Bigler J, Leppert M, Samowitz W, et al. (1999). "Meat consumption, genetic susceptibility, and colon cancer risk: a United States multicenter case-control study." Cancer Epidemiol Biomarkers Prev **8**(1): 15-24.

435 Jourenkova-Mironova N, Voho A, Bouchardy C, Wikman H, Dayer P,et al. (1999). "Glutathione S-transferase GSTM1, GSTM3, GSTP1 and GSTT1 genotypes and the risk of smoking-related oral and pharyngeal cancers." Int J Cancer **81**(1): 44-48.

436 García-Closas M, Kelsey KT, Hankinson SE, Spiegelman D, Springer K,et al. (1999). "Glutathione S-transferase mu and theta polymorphisms and breast cancer susceptibility." J Natl Cancer Inst **91**(22): 1960-1964.

437Gao, Y. and Q. Zhang (1999). "Polymorphisms of the GSTM1 and CYP2D6 genes associated with susceptibility to lung cancer in Chinese." Mutat Res **444**(2): 441-449.

438 Cheng L, Sturgis EM, Eicher SA, Char D, Spitz MR, et al. (1999). "Glutathione-S-transferase polymorphisms and risk of squamous-cell carcinoma of the head and neck." Int J Cancer **84**(3): 220-224.

439 Charrier J, Maugard CM, Le Mevel B, Bignon YJ.. (1999). "Allelotype influence at glutathione S-transferase M1 locus on breast cancer susceptibility." Br J Cancer **79**(2): 346-353.

440 Autrup JL, Thomassen LH, Olsen JH, Wolf H, Autrup H. (1999). "Glutathione S-transferases as risk factors in prostate cancer." Eur J Cancer Prev **8**(6): 525-532.

411 Ambrosone CB, Coles BF, Freudenheim JL, Shields PG. (1999). "Glutathione-S-transferase (GSTM1) genetic polymorphisms do not affect human breast cancer risk, regardless of dietary antioxidants." J Nutr **129**(2S Suppl): 565S-568S.

442 Abdel-Rahman SZ, Soliman AS, Bondy ML, Wu X, El-Badawy SA, et al. (1999). "Polymorphism of glutathione S-transferase loci GSTM1 and GSTT1 and susceptibility to colorectal cancer in Egypt." Cancer Lett **142**(1): 97-104.

443 Trizna Z, de Andrade M, Kyritsis AP, Briggs K, Levin VA, et al. (1998). "Genetic polymorphisms in glutathione S-transferase mu and theta, N-acetyltransferase, and CYP1A1 and risk of gliomas." Cancer Epidemiol Biomarkers Prev **7**(6): 553-555.

444 Saarikoski ST, Voho A, Reinikainen M, Anttila S, Karjalainen A, et al. (1998). "Combined effect of polymorphic GST genes on individual susceptibility to lung cancer." Int J Cancer **77**(4): 516-521.

445 Murata M, Shiraishi T, Fukutome K, Watanabe M, Nagao M, et al. (1998). "Cytochrome P4501A1 and glutathione S-transferase M1 genotypes as risk factors for prostate cancer in Japan." Jpn J Clin Oncol **28**(11): 657-660.

446 Maugard CM, Charrier J, Bignon YJ. (1998). "Allelic deletion at glutathione S-transferase M1 locus and its association with breast cancer susceptibility." Chem Biol Interact **111-112**: 365-375.

447 Lin DX, Tang YM, Peng Q, Lu SX, Ambrosone CB, et al. (1998). "Susceptibility to esophageal cancer and genetic polymorphisms in glutathione S-transferases T1, P1, and M1 and cytochrome P450 2E1." Cancer Epidemiol Biomarkers Prev **7**(11): 1013-1018.

448 Jourenkova N, Reinikainen M, Bouchardy C, Dayer P, Benhamou S, et al. (1998). "Larynx cancer risk in relation to glutathione S-transferase M1 and T1 genotypes and tobacco smoking." Cancer Epidemiol Biomarkers Prev **7**(1): 19-23.

449 Hong YS, Chang JH, Kwon OJ, Ham YA, Choi JH. (1998). "Polymorphism of the CYP1A1 and glutathione-S-transferase gene in Korean lung cancer patients." Exp Mol Med **30**(4): 192-198.

450 Hengstler JG, Kett A, Arand M, Oesch-Bartlomowicz B, Oesch F, et al. (1998). "Glutathione S-transferase T1 and M1 gene defects in ovarian carcinoma." Cancer Lett **130**(1-2): 43-48.

451 Helzlsouer KJ, Selmin O, Huang HY, Strickland PT, Hoffman S, et al. (1998). "Association between glutathione S-transferase M1, P1, and T1 genetic polymorphisms and development of breast cancer." J Natl Cancer Inst **90**(7): 512-518.

452 González MV, Alvarez V, Pello MF, Menéndez MJ, Suárez C, et al. (1998). "Genetic polymorphism of N-acetyltransferase-2, glutathione S-transferase-M1, and cytochromes P450IIE1 and P450IID6 in the susceptibility to head and neck cancer." J Clin Pathol **51**(4): 294-298.

453 González MV, Alvarez V, Pello MF, Menéndez MJ, Suárez C, et al. (1998). "Glutathione S-transferase GSTM1 and GSTT1 polymorphisms and colorectal cancer risk: a prospective study." Cancer Epidemiol Biomarkers Prev **7**(11): 1001-1005.

454 Benhamou S, Reinikainen M, Bouchardy C, Dayer P, Hirvonen A. et al. (1998). "Association between lung cancer and microsomal epoxide hydrolase genotypes." Cancer Res **58**(23): 5291-5293.

455 Bartsch H, Malaveille C, Lowenfels AB, Maisonneuve P, Hautefeuille A, et al. (1998). "Genetic polymorphism of N-acetyltransferases, glutathione S-transferase M1 and NAD(P)H:quinone oxidoreductase in relation to malignant and benign pancreatic disease risk. The International Pancreatic Disease Study Group." Eur J Cancer Prev **7**(3): 215-223.

456 Abdel-Rahman SZ, Anwar WA, Abdel-Aal WE, Mostafa HM, Au WW. (1998). "GSTM1 and GSTT1 genes are potential risk modifiers for bladder cancer." Cancer Detect Prev **22**(2): 129-138.

457 Wiencke JK, Wrensch MR, Miike R, Zuo Z, Kelsey KT. (1997). "Population-based study of glutathione S-transferase mu gene deletion in adult glioma cases and controls." Carcinogenesis **18**(7): 1431-1433.

458 To-Figueras J, Gené M, Gómez-Catalán J, Galán MC, Fuentes M, et al. (1997). "Glutathione S-transferase M1 (GSTM1) and T1 (GSTT1) polymorphisms and lung cancer risk among Northwestern Mediterraneans." Carcinogenesis **18**(8): 1529-1533.

459 Nimura Y, Yokoyama S, Fujimori M, Aoki T, Adachi W, et al. (1997). "Genotyping of the CYP1A1 and GSTM1 genes in esophageal carcinoma patients with special reference to smoking." Cancer **80**(5): 852-857.

460 Morita S, Yano M, Shiozaki H, Tsujinaka T, Ebisui C, et al. (1997). "CYP1A1, CYP2E1 and GSTM1 polymorphisms are not associated with susceptibility to squamous-cell carcinoma of the esophagus." Int J Cancer **71**(2): 192-195.

461 Kihara M, Kihara M, Kubota A, Furukawa M, Kimura H. (1997). "GSTM1 gene polymorphism as a possible marker for susceptibility to head and neck cancers among Japanese smokers." Cancer Lett **112**(2): 257-262.

462 Kelsey KT, Wrensch M, Zuo ZF, Miike R, Wiencke JK. (1997). "A population-based case-control study of the CYP2D6 and GSTT1 polymorphisms and malignant brain tumors." Pharmacogenetics **7**(6): 463-468.

463 Kelsey KT, Spitz MR, Zuo ZF, Wiencke JK. (1997). "Polymorphisms in the glutathione S-transferase class mu and theta genes interact and increase susceptibility to lung cancer in minority populations (Texas, United States)." Cancer Causes Control **8**(4): 554-559.

464 Kelsey KT, Hankinson SE, Colditz GA, Springer K, Garcia-Closas M, et al. (1997). "Glutathione S-transferase class mu deletion polymorphism and breast cancer: results from prevalent versus incident cases." Cancer Epidemiol Biomarkers Prev **6**(7): 511-515.

465 Jourenkova N, Reinikanen M, Bouchardy C, Husgafvel-Pursiainen K, Dayer P, et al. (1997). "Effects of glutathione S-transferases GSTM1 and GSTT1 genotypes on lung cancer risk in smokers." Pharmacogenetics **7**(6): 515-518.

466 Hung HC, Chuang J, Chien YC, Chern HD, Chiang CP, et al. (1997). "Genetic polymorphisms of CYP2E1, GSTM1, and GSTT1; environmental factors and risk of oral cancer." Cancer Epidemiol Biomarkers Prev **6**(11): 901-905.

467 Hori H, Kawano T, Endo M, Yuasa Y. (1997). "Genetic polymorphisms of tobacco- and alcohol-related metabolizing enzymes and human esophageal squamous cell carcinoma susceptibility." J Clin Gastroenterol **25**(4): 568-575.

468 Garcia-Closas M, Kelsey KT, Wiencke JK, Xu X, Wain JC, et al. (1997). "A case-control study of cytochrome P450 1A1, glutathione S-transferase M1, cigarette smoking and lung cancer susceptibility (Massachusetts, United States)." Cancer Causes Control **8**(4): 544-553.

469 Esteller M, García A, Martínez-Palones JM, Xercavins J, Reventós J. (1997). "Susceptibility to endometrial cancer: influence of allelism at p53, glutathione S-transferase (GSTM1 and GSTT1) and cytochrome P-450 (CYP1A1) loci." Br J Cancer **75**(9): 1385-1388.

470 El-Zein RA, Zwischenberger JB, Abdel-Rahman SZ, Sankar AB, Au WW. (1997). "Polymorphism of metabolizing genes and lung cancer histology: prevalence of CYP2E1 in adenocarcinoma." Cancer Lett **112**(1): 71-78.

471 Chen CL, Liu Q, Pui CH, Rivera GK, Sandlund JT,et al. (1997). "Higher frequency of glutathione S-transferase deletions in black children with acute lymphoblastic leukemia." Blood **89**(5): 1701-1707.

472 Brüning T, Lammert M, Kempkes M, Thier R, Golka K, et al. (1997). "Influence of polymorphisms of GSTM1 and GSTT1 for risk of renal cell cancer in workers with long-term high occupational exposure to trichloroethene." Arch Toxicol **71**(9): 596-599.

473 To-Figueras J, Gene M, Gomez-Catalan J, Galan C, Firvida J, et al. (1996). "Glutathione-S-Transferase M1 and codon 72 p53 polymorphisms in a northwestern Mediterranean population and their relation to lung cancer susceptibility." Cancer Epidemiol Biomarkers Prev **5**(5): 337-342.

474 Sarhanis P, Redman C, Perrett C, Brannigan K, Clayton RN, et al. (1996). "Epithelial ovarian cancer: influence of polymorphism at the glutathione S-transferase GSTM1 and GSTT1 loci on p53 expression." Br J Cancer **74**(11): 1757-1761

475 Moreira A, Martins G, Monteiro MJ, Alves M, Dias J, et al. (1996). "Glutathione S-transferase mu polymorphism and susceptibility to lung cancer in the Portuguese population." Teratog Carcinog Mutagen **16**(5): 269-274.

476 Katoh T, Nagata N, Kuroda Y, Itoh H, Kawahara A, et al. (1996). "Glutathione S-transferase M1 (GSTM1) and T1 (GSTT1) genetic polymorphism and susceptibility to gastric and colorectal adenocarcinoma." Carcinogenesis **17**(9): 1855-1859.

477 Kato S, Onda M, Matsukura N, Tokunaga A, Matsuda N, et al. (1996). "Genetic polymorphisms of the cancer related gene and Helicobacter pylori infection in Japanese gastric cancer patients. An age and gender matched case-control study." Cancer **77**(8 Suppl): 1654-1661.

478 Jahnke V, Matthias C, Fryer A, Strange R. et al. (1996). "Glutathione S-transferase and cytochrome-P-450 polymorphism as risk factors for squamous cell carcinoma of the larynx." Am J Surg **172**(6): 671-673.

479 Guo JY, Wan DS, Zeng RP, Zhang Q. (1996). "The polymorphism of GSTM1, mutagen sensitivity in colon cancer and healthy control." Mutat Res **372**(1): 17-22.

480 Anwar WA, Abdel-Rahman SZ, El-Zein RA, Mostafa HM, Au WW. (1996). "Genetic polymorphism of GSTM1, CYP2E1 and CYP2D6 in Egyptian bladder cancer patients." Carcinogenesis **17**(9): 1923-1929.

481 Yu MW, Gladek-Yarborough A, Chiamprasert S, Santella RM, Liaw YF, et al. (1995). "Cytochrome P450 2E1 and glutathione S-transferase M1 polymorphisms and susceptibility to hepatocellular carcinoma." Gastroenterology **109**(4): 1266-1273.

482 Trizna Z, Clayman GL, Spitz MR, Briggs KL, Goepfert H. (1995). "Glutathione s-transferase genotypes as risk factors for head and neck cancer." Am J Surg **170**(5): 499-501.

483 McGlynn KA, Rosvold EA, Lustbader ED, Hu Y, Clapper ML, et al. (1995). "Susceptibility to hepatocellular carcinoma is associated with genetic variation in the enzymatic detoxification of aflatoxin B1." Proc Natl Acad Sci U S A **92**(6): 2384-2387

484 London SJ, Daly AK, Cooper J, Navidi WC, Carpenter CL,et al. (1995). "Polymorphism of glutathione S-transferase M1 and lung cancer risk among African-Americans and Caucasians in Los Angeles County, California." J Natl Cancer Inst **87**(16): 1246-1253.

485 Kihara, M. and K. Noda (1995). "Distribution of GSTM1 null genotype in relation to gender, age and smoking status in Japanese lung cancer patients." Pharmacogenetics **5 Spec No**: S74-79.

486 Katoh T, Inatomi H, Nagaoka A, Sugita A. (1995). "Cytochrome P4501A1 gene polymorphism and homozygous deletion of the glutathione S-transferase M1 gene in urothelial cancer patients." Carcinogenesis **16**(3): 655-657.

487 Chenevix-Trench G, Young J, Coggan M, Board P. (1995). "Glutathione S-transferase M1 and T1 polymorphisms: susceptibility to colon cancer and age of onset." Carcinogenesis **16**(7): 1655-1657.

488 Zhong S, Wyllie AH, Barnes D, Wolf CR, Spurr NK. (1993). "Relationship between the GSTM1 genetic polymorphism and susceptibility to bladder, breast and colon cancer." Carcinogenesis **14**(9): 1821-1824.

489 Hirvonen A, Husgafvel-Pursiainen K, Anttila S, Vainio H. (1993). "The GSTM1 null genotype as a potential risk modifier for squamous cell carcinoma of the lung." Carcinogenesis **14**(7): 1479-1481.

490 Brockmöller J, Kerb R, Drakoulis N, Nitz M, Roots I. (1993). "Genotype and phenotype of glutathione S-transferase class mu isoenzymes mu and psi in lung cancer patients and controls." Cancer Res **53**(5): 1004-1011.
